# Long-Term Outcomes of Acute Osteoarticular Infections in Children

**DOI:** 10.3389/fped.2020.587740

**Published:** 2020-11-25

**Authors:** Nora Manz, Andreas H. Krieg, Michael Buettcher, Nicole Ritz, Ulrich Heininger

**Affiliations:** ^1^Infectious Diseases and Vaccinology Unit, University of Basel Children's Hospital, Basel, Switzerland; ^2^Orthopaedic Department, University of Basel Children's Hospital, Basel, Switzerland; ^3^Infectious Diseases Unit, Children's Hospital Lucerne, Lucerne, Switzerland

**Keywords:** osteomyelitis, septic arthritis, sequelae, outcome, children

## Abstract

**Background:** Acute hematogenous osteomyelitis (OM) and septic arthritis require immediate diagnosis and treatment by an interdisciplinary team of pediatric infectious disease specialists and pediatric orthopedic surgeons. Adverse outcomes such as growth disturbance, bone deformity, and chronic infections have been described in older studies. However, there is only little known about long-term follow-up of patients of the last two decades. Therefore, we aimed to evaluate subjective and objective long-term outcomes of these children with osteoarticular infections treated in the millennial years.

**Methods:** Cross-sectional study performed in two pediatric centers including patients admitted for OM and/or SA between 2005 and 2014 and follow-up consultations in 2019. Patients with symptoms of ≤2 weeks duration at initial presentation were contacted. Subjective outcomes were assessed by standardized interview, objective outcomes by clinical examination. Medical charts were used to extract data from the initial presentations. Statistical analysis was performed by non-parametric tests and Fisher's exact test.

**Results:** Of 147 eligible patients 77 (52%) agreed to participate, of which 68 (88%) had an interview and physical examination and 9 (12%) an interview only. Thirty-three (39%) had OM, 26 (34%) SA, and 21 (27%) combined OM/SA. Median (IQR) age at follow-up was 13.3 (10.5–18.0) years with a median (IQR) follow-up of 7.1 (6.1–8.6) years. Persistent complaints including pain, functional differences and scar paresthesia, reported by 21 (28%) patients, were generally mild and only 3 (5%) required ongoing medical care. Objective sequelae including pain, limited range of motion, unilateral axis deformity or asymmetric gait were found in 8 (12%) participants. Older age, female sex, joint involvement, surgical intervention, persistent fever, and C-reactive protein elevation were associated with adverse clinical outcome.

**Conclusions:** Adverse outcomes were observed in a considerable number of patients, most of which were minor, and only few required ongoing medical care. Long-term follow up is advisable for patients with risk factors identified during the initial presentation.

This study was registered on ClinicalTrials.gov (NCT03827980).

## Introduction

Acute hematogenous osteomyelitis (OM) and septic arthritis (SA) in children are severe diseases with yearly incidence rates between 0.5 and 11 per 100'000 (1–3). Children usually present within 2 weeks after onset of pain and signs of inflammation at the affected limb (4, 5). For optimal management of OM and SA, immediate interdisciplinary management by pediatric infectious disease specialists and pediatric orthopedic surgeons is crucial.

Long-term sequelae such as growth disturbance, bone deformity, and chronic infection have been described in 2–40% of patients (6–11). Septic arthritis of the hip seems to be especially prone for long-term sequelae, which were reported in 47% of affected newborns and in 66% of affected children in a South African study (12, 13). Other factors that have been associated with poor long-term outcome are young age, infection with Methicillin-resistant *Staphylococcus aureus* (MRSA), and delay of appropriate treatment initiation (7, 13–16).

Previous studies on long-term outcomes of OM and SA mostly referred to pre-millennial patient cohorts. During the last two decades, management of osteoarticular infections in children has advanced with increasing use of magnetic resonance imaging (MRI), microbial testing (i.e., nucleic acid amplification and resistance testing) and recommendations for shorter treatment duration (17–23). Clearly, long-term outcome studies evaluating modern management are needed. Therefore, we aimed to evaluate subjective and objective long-term outcomes of children with previous OM and SA.

## Materials and Methods

### Study Design, Setting, and Sample

This was a cross-sectional study performed at two pediatric centers in central Europe. Children who had been admitted between 2005 and 2014 with an international classification of disease discharge diagnosis of osteomyelitis and/or septic arthritis (International classification of diseases 10th revision, ICD-10 M00.00-00.99 and/or M86.00-M86.99) were screened for eligibility based on medical records by the following inclusion criteria: history of symptoms of ≤ 2 weeks duration on admission, no penetrating wound or prior surgery at the affected location, no chronic or severe underlying disease, and correct ICD-10 coding.

Eligible patients were contacted by letter and by a phone call between February 1st and May 15th 2019. In case the letter was returned to sender or no valid phone numbers were available, we contacted the patient's family doctor and asked to forward the information letter to the patient. If patients or their care-givers had insufficient German language skills to consent to the study, they were not included. All interested patients received an appointment for a follow-up study visit between March 6th and May 31st 2019. Data from the initial disease were extracted from medical records (Supplementary Material 1).

### Study Procedures

Participation in the study consisted of a single follow-up visit with a standardized interview (Supplementary Materials 2, 3) and a physical examination (Supplementary Material 4). Participants who did not agree to a visit for a physical examination were asked to participate in a phone interview. The interview included subjective health assessments by the participants, such as rating the outcome of their prior OM and/or SA with a health grade from 1 (poor outcome) to 5 (excellent outcome). If participants rated the outcome below 5, they were asked to describe their complaints. In addition, participants were asked to assess their capacities for various daily activities of the previously affected extremity. To quantify functional disabilities, we modified the questions and score scale of the “Pediatric Outcomes Data Collection Instrument” (PODCI) to our needs (24, 25) (Supplementary Material 5). “Subjective sequelae” were defined as a health grade <5 or a moderate or severe disability score. Participants who were older than 7 years at time of diagnosis were asked to report their sport activities at three different time points: before diagnosis, 1 year after discharge, and at follow-up.

Physical examination included assessment of limb axis and limb length, range of motion of the affected and/or adjacent joints, as well as observation of gait in case the lower extremity was initially affected (Supplementary Material 4). Standardized photographic and video documentation was done for limb axis and gait assessments. “Objective sequelae” were defined as any of the following observations: limb length discrepancy of > 1 cm, pain during examination, functional deficit in range of motion of > 20° compared to the contralateral side, asymmetric gait, or asymmetric limb axis. All study procedures were performed by a single, trained investigator (NM). Photographic and video material was independently analyzed by a pediatric orthopedic specialist blinded to the outcome (AHK).

### Data Management and Statistical Analyses

All data were entered into an electronic case report form (Secu Trial, Version 5.5.1.10, 2019 © 2000–2019 interActive Systems, Berlin, Germany). We used non-parametric tests to compare groups and Fisher's exact test to compare ratios. Statistical analysis were performed using RStudio (Version 1.1.463–© 2009–2018 RStudio, Inc., Boston, USA).

### Ethics

Informed consent was obtained from all participants and their legal guardians. The study was approved by the local ethics committee of North-Western Switzerland (EKNZ 2018-02393) and this study was registered on ClinicalTrials.gov (NCT03827980).

## Results

### Baseline Characteristics of Study Population

**Figure 1:**
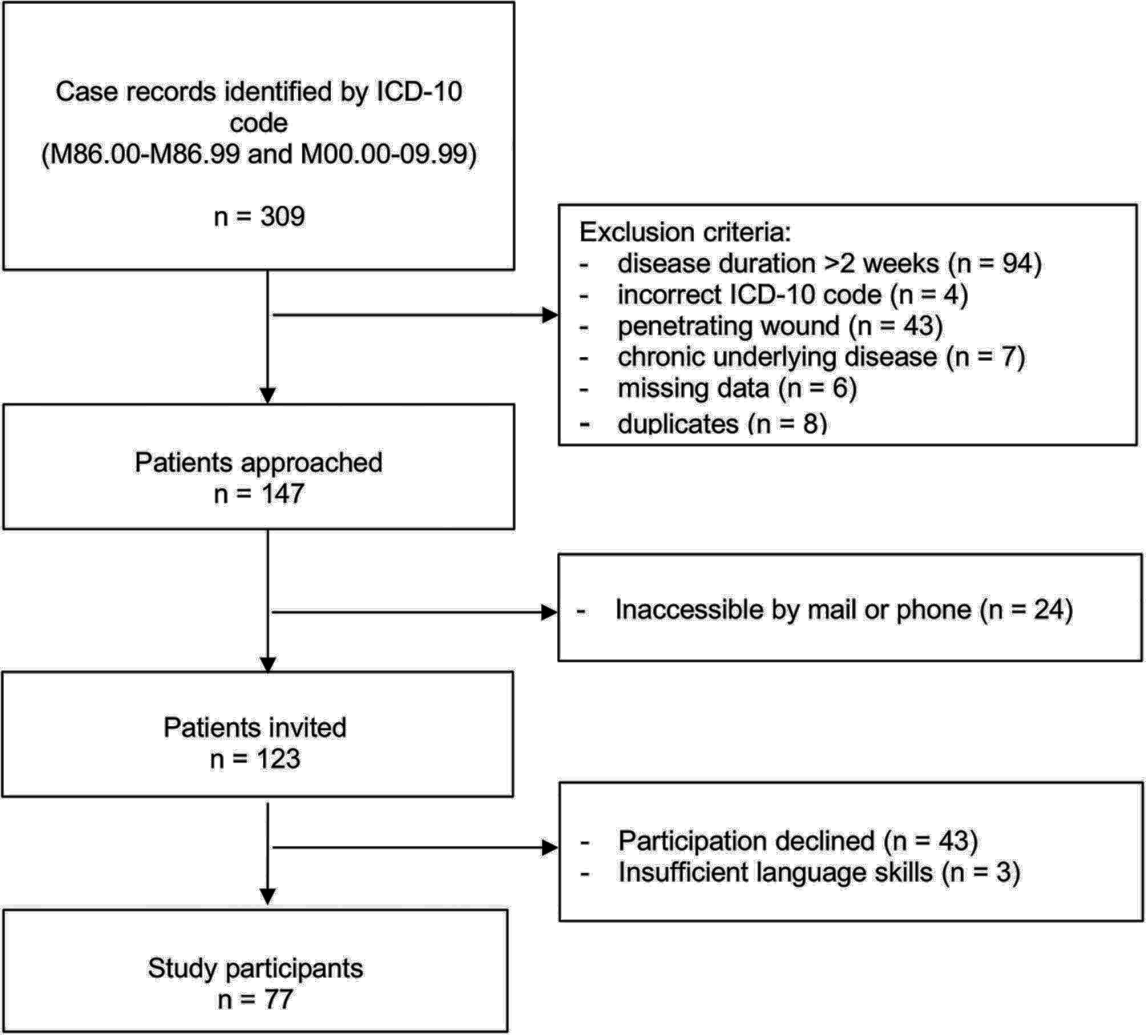
Flow chart of recruitment.

Of 309 screened case records, 147 fulfilled eligibility criteria; of those 77 (52%) agreed to participate in the study (Figure 1). Sixty-eight (88%) had an interview and physical examination and 9 (12%) an interview only. Median (IQR) age was 5.9 (1.9–10.8) years at time of initial admission and 13.3 (10.5–18.0) years at follow-up. Follow-up time ranged from 4.5 to 14.5 years with a median (IQR) follow-up time of 7.1 (6.1–8.6) years. A total of 32 (42%) patients were female. Thirty (39%) patients had OM, 26 (34%) SA, and 21 (27%) combined OM and SA. The infected bone or joint was in the lower extremity or pelvic region in 66 (86%) of cases (Table 1).

**Table 1:** Characteristics of initial disease in study participants by presence or absence of sequelae on follow-up.

	**All**	**Subjective sequelae**			**Objective sequelae**		
**General characteristics**	***n* = 77**	**Yes (*n* = 21/77)**	**No (*n* = 56/77)**	***p***	**Yes (*n* = 8/68)**	**No (*n* = 60/68)**	**p-value**
*N* females (%)	32 (42%)	13 (62)	19 (34)	0.04	6 (75)	22 (37)	0.06
Age at study visit [years]; median (IQR)	13.3 (10.5–18.0)	16.4 (15.3–19.3)	11.5 (9.4–17.6)	0.006	13.9 (10.1–17.6)	13.3 (10.4–18.2)	0.80
Age on admission [years]; median (IQR)	5.9 (1.9–10.8)	8.9 (4.7–10.8)	4.0 (1.5–10.3)	0.05	6.6 (3.2–10.1)	6.2 (2.1–10.7)	0.98
Follow-up time [years]; median (IQR)	7.1 (6.1–8.6)	7.6 (7–10.8)	6.9 (6.0–8.3)	0.06	7 (6.5–7.7)	7.1 (6.2–8.7)	0.85
Joint involvement (SA or OM + SA), N (%)	47 (61)	16 (76)	31 (55)	0.12	8 (100)	34 (57)	0.02
Duration of complaint [days]; median (IQR)	3 (1.75–7)	3 (3–7)	3 (1–6.5)	0.79	3 (1–3)	3 (2–7)	0.23
Duration of admission [days], median (IQR)	14 (11–15)	14 (10–18)	13.5 (11–15)	0.23	15.5 (13.0–18.8)	13.0 (11.0–15.0)	0.20
**Location of infection**							
Upper limb; *N* (%) Shoulder region; *N* (%) Elbow region; *N* (%) Wrist region; *N* (%) Lower limb; *N* (%) Hip /pelvic region; *N* (%) Sacroiliac joint; *N* (%) Knee region; *N* (%) Central lower leg; *N* (%) Talocrural joint region; *N* (%) Foot region; *N* (%)	11 (14) 6 (8) 4 (5) 1 (1) 66 (86) 13 (17) 2 (3) 29 (38) 2 (3) 11 (14) 9 (12)	3 (14) 1 (5) 1 (5) 1 (5) 18 (86) 3 (14) 1 (5) 8 (38) 1 (5) 5 (23) 0 (0)	8 (14) 5 (8) 3 (5) 0 (0) 48 (86) 10 (18) 1 (2) 20 (36) 1 (2) 7 (13) 9 (16)	1.0 1.0 1.0 0.27 1.0 1.0 0.47 1.0 0.47 0.29 0.10	1 (12) 0 (0) 1 (12) 0 (0) 7 (88) 3 (38) 0 (0) 3 (38) 0 (0) 1 (12) 0 (0)	9 (15) 5 (8) 3 (5) 1 (2) 51 (85) 10 (17) 2 (3) 20 (33) 2 (3) 10 (17) 7 (11)	1.0 1.0 0.40 1.0 1.0 0.17 1.0 1.0 1.0 1.0 0.59
**Laboratory tests performed**							
WBC; *N* (%)	77 (100)	21 (100)	56 (100)		8 (100)	60 (100)	
WBC [× 10^9^/l]; median (IQR)	10.8 (7.5–14.2)	9.9 (8.9–12.9)	11.4 (7.4–14.3)	0.57	10.7 (9.4–12.9)	10.5 (7.4–14.2)	0.93
Neutrophil band count; *N* (%)	54 (70)	14 (67)	40 (71)		7 (88)	38 (63)	
Neutrophil band count [%]; median (IQR)	6 (3.25–17.75)	6.5 (0.3–21.3)	6 (4.0–16.3)	0.66	7.0 (4.0–29.5)	5.0 (1.3–15.0)	0.45
ESR [mm/h]; median (IQR)	54 (35–75)	44 (30–68)	60 (38–75)	0.25	47 (32.5–81.5)	54 (32–75)	0.88
CRP on admission; *N* (%)	76 (99)	20 (95)	56 (100)		8 (100)	59 (98)	
CRP [mg/l]; median (IQR)	49.5 (21.5–104.75)	59 (25–111)	46 (21–98)	0.70	85.5 (33.8–111.5)	49 (17–99.5)	0.28
CRP 24–72 h after admission; *N* (%)	56 (73)	19 (90)	37 (66)		8 (100)	39 (65)	
CRP [mg/l]; median (IQR)	57.5 (23–131.25)	100 (32–136)	48 (23–120)	0.32	131.5 (71.0–138.3)	53.0 (28.5–131.5)	0.33
CRP 72–120 h after admission; *N* (%)	48 (62)	14 (67)	34 (61)		7 (88)	38 (63)	
CRP [mg/l]; median (IQR)	27 (9–83.75)	77 (59–137)	21 (7–44)	0.006	146.0 (70.0–157.5)	24.5 (9.0–68.3)	0.02
Blood culture; *N* positive/*N* total (%)	18/65 (28)	5/19 (26)	13/46 (28)	1.0	3/8 (38)	14/50 (28)	0.68
Causative organism found; *N* (%)^2^	41/77 (53)	13/21 (62)	28/56 (50)	0.45	6/8 (75)	29/60 (48)	0.26
**Observations during hospital stay**							
Fever > 48 h after first dose; *N* (%)	19 (25)	12 (57)	7 (13)	<0.001	6 (75)	12 (20)	0.003
ICU admission; *N* (%)	5 (6)	1 (5)	4 (7)	1.0	1 (13)	3 (5)	0.40
Disseminated disease; *N* (%)	1 (1)	1 (5)	0 (0)	0.27	0 (0)	1 (2)	1.0
**Intervention/Treatment**							
Surgical procedures performed; median (IQR)	1 (1–2)	1 (1–2)	1 (0.75–1)	0.02	1.5 (1.0–2.25)	1 (1–1)	0.03
Surgical procedures performed; *N* (%) One; *N* (%) Two; *N* (%) Three; *N* (%) Four; *N* (%) Five; *N* (%)	62 (81) 45 (72) 11 (18) 2 (3) 3 (5) 1 (2)	20 (95) 13 (65) 3 (15) 1 (5) 2 (10) 1 (5)	42 (75) 32 (76) 8 (19) 1 (2) 1 (2) 0	0.06 0.38 1.0 0.55 0.24 0.32	8 (100) 4 (50) 2(25) 1 (13) 0 (0) 1 (13)	46 (77) 33 (72) 9 (20) 1 (2) 3 (6) 0 (0)	0.19 0.24 0.66 0.28 1.0 0.15
Duration of i.v. antibiotic treatment [days], median (IQR)	13 (11–15)	15 (11–17)	13 (10.8–15.0)	0.29	15 (12–15.5)	13 (11–15)	0.38
Duration of overall antibiotic treatment [days], median (IQR)	42 (37–44.5)	43 (35.5–44.5)	42 (37–44.3)	0.71	43.5 (39.5–49)	42 (37–45)	0.51

*IQR, Interquartile range; WBC, white blood cell; CRP, C-reactive protein; ESR, Erythrocyte sedimentation rate; i.v., intravenous*.

Five (6%) participants had been readmitted due to complaints associated with the initially affected body part. Readmissions were required within 1 week (bone sequester), 1 month (arthroscopic adhesiolysis and refusal to walk, respectively), 2 years (talocrural joint revision), and 12 years (resection of meniscal cyst) after discharge.

None of the participants experienced a relapse of infection as defined by positive cultures from tissue or blood.

### Subjective Sequelae

Seventy-six (99%) participants reported that they recalled the admission for an osteoarticular infection. Participants reported to have fully recovered 1 week (*n* = 14; 18%), 1 month (*n* = 17; 22%), within 5 months (*n* = 29; 38%), 6 months (*n* = 2; 3%), or 12 months (*n* = 5; 6%) after admission to hospital; 4 (5%) participants did not remember. Two participants (3%) reported that recovery took several years, and 4 (5%) reported that they have not yet fully recovered, 3 of them requiring ongoing medical care. Most participants (*n* = 56, 73%) rated the outcome of the previously affected limb as excellent with health grade 5. A total of 21 participants assigned a health grade between 4 and 4.75 (*n* = 15), 3–3.75 (*n* = 5), or a grade of 2 (*n* = 1). Their reported limitations varied from pain to limited range of motion and are summarized in detail in Table 2.

**Table 2:** Characteristics of participants with subjective and/or objective sequelae.

**Health grade**	**Complaint**	**Frequency of pain**	**Examination findings on follow-up**	**Sex**	**Age at follow-up (in years)**	**Age on admission (in years)**	**Type of infection**	**Surgical interventions**	**Readmission**
**Subjective sequelae (*****n*** **=** **14)**									
3	Difference in function	Never	Normal	Male	14.3	7.9	OM	0	No
4	Pain	More than once a week	Normal	Female	20.9	12.3	OM	1	No
4	Pain	Once a week	Normal	Female	15.3	2.9	SA	1	No
4	Scar paresthesia	Once a week	Normal	Female	15.4	9.6	OM+SA	1	No
4	Scar paresthesia	Never	Normal	Female	15.6	4.7	OM	1	No
4	Caution	Never	Normal	Female	24.9	12.3	OM	1	No
4	Pain	More than once a week	Normal	Male	15.9	6.6	OM+SA	1	No
4	Difference in function	Never	Normal	Male	19.5	13.9	OM	1	No
4.5	Odd feeling	Never	Normal	Female	12.3	1.5	SA	2	No
4.5	Pain	Less than once month	Normal	Male	19.3	6.8	SA	4	No
4.5	Pain	Less than once month	Normal	Male	23	8.9	SA	4	No
4.5	Pain	Once a month	Normal	Female	18.9	11.9	SA	1	No
4.5	Pain	Less than once month	Normal	Male	17.8	10.7	OM+SA	1	No
4.75	Odd feeling	Never	Normal	Female	16.3	9.1	SA	3	No
**Subjective and objective sequelae (*****n*** **=** **7)**									
2	Pain	Daily	Pain during examination	Female	18.9	11.9	OM+SA	2	Yes to another hospital
3	Difference in function	Never	Limited range of motion, asymmetric arm axis	Female	8.9	0.8	SA	1	No
3	Pain	Daily	Limited range of motion, pain during examination	Female	16.4	8.8	OM+SA	1	No
3.5	Difference in function	Never	Asymmetric gait, pain during hip exam	Female	11.3	4.3	OM+SA	5	No
3.5	Pain	Once a month	Pain during examination	Male	17.1	10.8	SA	1	No
4	Caution	Never	Asymmetric leg axis	Male	6.1	0.9	SA	1	No
4	Difference in function	Never	Asymmetric leg axis	Female	20	9.8	OM+SA	2	No
**Objective sequelae (*****n*** **=** **1)**									
5	None	Never	Limited range of motion	Female	10.5	4	OM+SA	3	No

Fifty-seven (73%) participants reported to be fully competent with daily activities, 18 (23%) had minor disabilities 2 (3%) had moderate disabilities, and none reported severe disability. Sixteen (76%) of 21 participants with health grade <5 also reported minor or moderate disabilities.

Sport activities before admission, after discharge and at follow-up at a frequency of ≥ 2 times per week were reported by 52, 51, and 61%, once a week in 30, 27, and 9%, and once a month or less in 18, 21, and 30% of participants, respectively. Reported sport activities, when compared between before and 1 year after the osteoarticular infection, had remained the same in 76%, increased in 9%, and declined in 15% of participants. At follow-up, sport activities had remained the same in 29%, increased in 38%, and declined in 32% compared to before onset of the osteoarticular infection. All six participants who had performed sports on a competitive level before onset of the osteoarticular infection discontinued to do so. Reasons for discontinuation were recurrent pain in two participants and loss of interest in four participants.

### Objective Sequelae

Objective sequelae were observed in 8 (12%) participants and involved one or more of the following: pain during examination (*n* = 4), a deficit in range of motion of ≥ 20° (*n* = 3), unilateral axis deformity (*n* = 3: varus of the knee, valgus of the knee, valgus of the elbow), and asymmetric gait with predominant internal rotation of the affected hip (*n* = 1).

Physical examination was normal in 14 (66%) of 21 participants with health grade <5.

Median subjective health grade was lower in the eight participants with objective sequelae than in 60 participants without objective sequelae (3.5; IQR 3–4 vs. 5; IQR 5-5, *p* < 0.001). Leg length difference of > 1 cm was not observed.

### Outcome Based on Characteristics of Initial Disease

#### Age and Disease Manifestations

Participants with subjective sequelae were older at time of study visit (median 16.4 years) than participants without complaints (median 11.5 years, *p* = 0.006). In participants with objective sequelae, joint involvement was 100% compared to 57% in those without objective sequelae (*p* = 0.02) (Table 1). Females had higher rates of subjective or objective sequelae compared to males (40 vs. 18%, *p* = 0.04, and 21 vs. 5%, *p* = 0.06, respectively).

#### Biomarkers and Surgical Interventions

C-reactive protein (CRP) values obtained 72–120 h after admission were higher, and persistence of fever beyond 48 h after the first antibiotic dose was more frequent in participants with compared to those without subjective and objective sequelae on follow-up (Table 1).

Overall, 90 surgical procedures were performed in 62 participants. Of these, 70 were in children with septic arthritis with or without osteomyelitis. These surgeries involved 63 joint lavages and 10 curretages of the adjacent bone, none of them bedside but all in theater. Interventions of the knees and talocrural joint were performed arthroscopically whereas both arthroscopies and arthrotomies were performed in hips. Specifically, 81 (90%) procedures were for collection of material for pathogen detection and were combined with lavage in 77 (86%), curettages in 27 (30%), and drainage placement in 17 (19%) of procedures. Material from the synovia were obtained by punch biopsies and no synovectomies were performed. Participants with subjective or objective sequelae had undergone more surgical interventions on average than those without (*p* = 0.02 and 0.03, respectively, Table 1).

#### Pathogens

A causative pathogen had been identified in 41 (53%) participants, either by blood culture or culture of material from the affected bone or joint. Main organisms found were *Staphylococcus aureus* (*n* = 27, including one case of Methicillin resistant *S. aureus*), *Streptococcus pyogenes* (*n* = 7), and *Streptococcus pneumoniae* (*n* = 5). Pathogen detection was not associated with sequelae (Table 1).

#### Antibiotic Treatment

Antibiotics had been administered intravenously for a median (IQR) of 13 (11–15) days. Data on overall treatment duration was available in 76 participants and showed a median (IQR) treatment duration of 42 (37–46) days. Treatment duration did not differ by diagnosis (OM, SA, or combined OM/SA). Most commonly used oral drugs were amoxicillin/clavulanic acid in 46 (59%) and clindamycin in 14 (18%) of participants. We did not observe differences in duration of treatment in participants with or without sequelae (Table 1).

## Discussion

To our knowledge, this is the first study that evaluates subjective and objective long-term outcomes in children with acute bone and joint infections treated in the last two decades. After a median of 7 years follow-up 73% of participants had an excellent outcome with no physical limitations reported and 88% had normal findings on follow-up clinical examination.

Several other studies have evaluated the outcome after osteoarticular infections in children but most of them focused on specific subgroups. A Korean study including children with hip arthritis <18 months of age observed excellent or good clinical outcomes in 84% after a mean follow-up of 6 years (26). Another study in 52 Australian children with septic arthritis reported normal function with excellent outcomes assessed by radiographic imaging in 81% of those with hip and 100% of those with knee arthritis after a mean follow-up of 8.5 years (11). A further study from the United States of America, including 134 children with acute or subacute osteomyelitis, reported excellent or good outcomes with complete clinical resolution and minor radiological changes assessed in 92% after a follow-up of 2.5 years (6).

Importantly, 27% of participants in this study reported some degree of persistent health limitations which could only partly be confirmed by clinical examination. This discrepancy may be due to the fact that some complaints such as scar paresthesia, apprehension, or intermittent pain are not detected during clinical examination.

Interestingly, participants who reported suboptimal outcomes (health grade <5) were older on admission and older at follow-up than those reporting excellent outcomes. This is in contrast to earlier studies which did not report a correlation between age and sequelae (8, 26). It is also in contrast to studies which reported young age, i.e., below 1 month (7) and 3 years (6), respectively, as a risk factor for adverse outcomes. A possible explanation for our findings is that children who had been at or beyond school age during their osteoarticular infections might experience and describe their health situation more precisely and nuancedly than younger children.

Both subjective and objective sequelae were more frequently described in female participants. This was not observed in other follow-up studies of osteoarticular infections (8, 26), and in several studies sex was not investigated as a risk factor for long-term sequelae (6, 7, 9, 10). However, current literature in the field of general orthopedics suggests that women experience more joint-related pain (27, 28).

We found that bone infections with joint involvement and septic arthritis were associated with more objective sequelae than bone infections alone. This is plausible, because in the inflamed joint, the cartilage and the epiphyseal plate of neighboring bones are at risk for structural damage. This can result in growth disturbance, pain, and altered range of motion due to fibrosis of the synovial capsule (29, 30). In other follow-up studies, which also included children with OM and/or SA, joint involvement as a risk factor was not assessed (7, 10). Other outcomes studies included children with either OM or SA and therefore could not assess joint involvement as a risk factor for adverse long-term outcomes (6, 9, 11, 26). The impact of age at onset of illness and the associated skeletal maturity as well as joint involvement as risk factors for adverse outcome of osteoarticular infections should be assessed in a future, prospective study.

CRP and duration of fever were associated with both subjective and objective sequelae, suggesting that the initial grade and duration of inflammation is a marker of tissue damage and later potential sequelae.

Risk for subjective or objective sequelae did not depend on the pathogen that had caused OM or SA. However, numbers are too small to draw any firm conclusions, and in only one of our patients was infection caused by MRSA, an organism previously shown to be a risk factor for severe sequelae (6, 7).

In previous studies mean duration of symptoms varied between 4.2 and 27 days and delay in treatment was associated with unfavorable outcomes (6–8, 26, 29). In contrast, duration of complaints before admission and initiation of treatment was similarly short in participants with or without sequelae in our study. This probably explains why duration of symptoms was not a risk factor for adverse outcome.

In our study cohort, both intravenous and overall treatment durations were rather long, with a median of 13 and 42 days, respectively. This is somewhat surprising since there have been studies performed lately that observed comparable clinical short-term outcomes with reduced treatment duration (22, 23). However, our cohort of patients was treated between 2005 and 2014, i.e., before recommendations for shorter antibiotic treatment were introduced on a national level in 2017 (31). Participants with both subjective and objective sequelae underwent more surgical interventions than those without sequelae, however this difference is of limited magnitude and whether this represents a risk factor for adverse outcome or is an indicator for a more severe course of disease remains unclear and should be investigated in future studies.

Our study has potential limitations. First, we assessed objective outcomes by physical examination only. This is in contrast with other studies in which diagnostic imaging was performed (6, 7, 11, 26). We were concerned that performing diagnostic imaging routinely as part of the follow-up study procedures would not be acceptable for many participants and lead to a selection bias in favor of those with ongoing complaints. Second, our modified PODCI score was not validated.

Third, when participants are enrolled retrospectively and patients with insufficient German language skills are excluded, selection bias should be considered. Yet, study participants were comparable to our previous retrospective study regarding age, gender, and location of infection, which suggests that we have studied a representative sample (21).

In summary, we observed adverse subjective and objective outcomes in a considerable number of patients. Most of those were minor and only few required ongoing medical care. Of note, this study was performed in a well-advanced health care system and therefore the results are not necessarily applicable in other health care systems.

We identified older age, female sex, joint involvement, number of surgical interventions, persistent fever, and CRP elevation as risk factors for adverse outcomes. Long-term follow up is advisable for patients with these risk factors.

## Data Availability Statement

The raw data supporting the conclusions of this article will be made available by the authors, without undue reservation.

## Ethics Statement

The studies involving human participants were reviewed and approved by ethics committee of North-Western Switzerland (EKNZ 2018-02393). Written informed consent to participate in this study was provided by the participants' legal guardian/next of kin.

## Author Contributions

NM recruited the participants, collected data, carried out the statistical analyses, and drafted the manuscript. AK analyzed the collected photo and video material of the study participants. MB supervised NM's work at the Children's Hospital Lucerne study site and provided the institutional resources. UH supervised NM's overall work, acquired additional funding, and provided the necessary institutional resources. NM wrote the first draft of the manuscript and all other authors revised it critically. All authors were involved in the design and planning of the study.

## Conflict of Interest

The authors declare that the research was conducted in the absence of any commercial or financial relationships that could be construed as a potential conflict of interest.

## Abbreviations

OM: osteomyelitis
SA: septic arthritis
CRP: C-reactive protein
IQR: interquartile range.
