# Supplementary material for: Long-Term Outcomes of Acute Osteoarticular Infections in Children
Source: Front Pediatr. 2020 Nov 25;8:587740. doi: 10.3389/fped.2020.587740 (PMC7737431; doi:10.3389/fped.2020.587740)
Supplement: Supplementary file 1 [file Data_Sheet_1.PDF]

Minimal Data Set mnpukb08min_data_set

{ IASAlternateStandardGroup: .. Standard - vertical, alternative layout }
Case Type

Query

Case Type

- OM (Osteomyelitis) (1 - 101765)
- SA (Septic Arthritis) (2 - 101766)
- coded OM + SA (3 - 102318)
- OM with signs of joint involvement (4 - 102319)
- SA with signs of bone involvement (5 - 102320)
- unconclusive (9 - 105011)

case_type {Horizontal Radiobutton}

{ IASAlternateStandardGroup: .. Standard - vertical, alternative layout }
Hospital

Query

Date of Admission

_ - _ - _ dd.mm.yyyy

date_admission {Date (dd.mm.yyyy), Date format: display, Missing Value}

Date of Discharge

_ - _ - _ dd.mm.yyyy

date_discharge {Date (dd.mm.yyyy), Date format: display, Missing Value}

Age at admission

_ . _

age_admission {Number 2,1}

{ IASAlternateStandardGroup: .. Standard - vertical, alternative layout }
Presentation

Query

Body temperature on admission

_ . _

temp_admission {Number 2,1}

{ IASTableRepetitionGroup: .... Tabular repetition group , Initial count: 1, Max. count: unlimited, Show "Delete" button: yes, Display delete confirmation: yes, Reverse repetitions: no, Initially ordered by: Displayed order }
height

Body height emnpukb08height

{ IASHorizontalGroup: .... Standard - horizontal }
height

No.	Date	Height	
1	_ - _ - _ dd.mm.yyyy<br>date_height {Date (dd.mm.yyyy), Date format: display, Missing Value}	_ . _ cm<br>height {Number 3,1}	Query

More

{ IASAlternateStandardGroup: .. Standard - vertical, alternative layout }
Duration of symptoms before admission

Query

Duration of symptoms before admission

_

symptom_dur_before {Number 2,0}

{ IASAlternateStandardGroup: .. Standard - vertical, alternative layout }
Site of infection

Query

Site of infection

one bone

one_bone {Checkbox}

one joint

one_joint {Checkbox}

one osteoarticular

one_osteoarticular {Checkbox}

two locations

two_locations {Checkbox}

three locations

three_locations {Checkbox}

more than 3 locations

more_3_locations {Checkbox}

{ IASTableRepetitionGroup: .... Tabular repetition group , Initial count: 1, Max. count: unlimited, Show "Delete" button: yes, Display delete confirmation: no, Reverse repetitions: no, Initially ordered by: Displayed order, Hide headline }

infection

bones details ***emnpukb08bones***

{ IASHorizontalGroup: .... Standard - horizontal }

bone locations

No.	Localisation of infection	specify other	
1	< Please choose ><br>loc_of_inf_bone {Popup (Label Group), Missing Value}	loc_inf_bone_other {Textfield 30}	Query

More

{ IASAlternateStandardGroup: .. Standard - vertical, alternative layout }

Source of information

Query

Source of information

letter

letter {Checkbox}

imaging findings

imaging_findings {Checkbox}

inconclusive/contradictory results

inconclusvie_res {Checkbox}

{ IASAlternateStandardGroup: .. Standard - vertical, alternative layout }

Febrile days under antibiotic treatment

Query

Febrile days under antibiotic treatment

[ ] days

frebile_antibiotics {Number 2,0}

{ IASAlternateStandardGroup: .. Standard - vertical, alternative layout }
ICU-admission

Query

ICU-admission

( ) ICU admission (1 - 102321) ( ) no ICU admission (0 - 102322)

icu_admission {Horizontal Radiobutton} "ICU-admission"

{ IASAlternateStandardGroup: .. Standard - vertical, alternative layout }
Disseminated Disease

Query

Disseminated Disease

( ) yes (1 - 101864) ( ) no (2 - 101747)

disseminated_disease {Horizontal Radiobutton}

{ IASAlternateStandardGroup: .. Standard - vertical, alternative layout }
Disseminated Disease

Query

Deep Venous Thrombosis

[ ]

dissem_thrombosis {Checkbox}

septic pulmonary embolism

[ ]

dissem_pulmon {Checkbox}

pneumonia

[ ]

dissem_pneumonia {Checkbox}

endocarditis

[ ]

dissem_endocarditis {Checkbox}

multi-focal infection

[ ]

dissem_multi_inf {Checkbox}

{ IASHeadline: .... Headline }

Laboratory

{ IASAlternateStandardGroup: .. Standard - vertical, alternative layout }
LABORATORY

Query

ESR on admission

[ ] mm/h

esr_admission {Number 3,0}

WBC admission

[ ] . [ ] x 10x9

wbc_admission {Number 2,1}

Band (WBC) count on admission

%
band_wbc_admission {Number 2,0}

{ IASTableRepetitionGroup: .... Tabular repetition group , Initial count: 1, Max. count: unlimited, Show "Delete" button: yes, Display delete confirmation: yes, Reverse repetitions: yes, Initially ordered by: Displayed order, Hide headline }
CRP
CRP emnpukb08crp
{ IASHorizontalGroup: .... Standard - horizontal }
crp
More

No.	Date	CRP	
1	- - dd.mm.yyyy<br>date_crp {Date (dd.mm.yyyy), Date format: display, Missing Value}	crp {Number 3,0}	Query

{ IASAlternateStandardGroup: .. Standard - vertical, alternative layout }
CRP
Query

CRP day 0 = admission

crp_day0 {Number 3,0}

{ IASHorizontalGroup: .... Standard - horizontal }
CRP hour 24-72
Query

CRP hour 24-72

Date	CRP
- - - - dd.mm.yyyy hh:mm<br>date_crp_24_72 {Checked Date (dd.mm.yyyy hh:mm), Date format: display, Missing Value}	crp_24_72 {Number 3,0, Missing Value}

{ IASHorizontalGroup: .... Standard - horizontal }
CRP hour 72-120
Query

CRP hour 72-120

Date	CRP
- - - - dd.mm.yyyy hh:mm<br>date_crp_72_120 {Checked Date (dd.mm.yyyy hh:mm), Date format: display, Missing Value}	crp_72_120 {Number 3,0, Missing Value}

{ IASAlternateStandardGroup: .. Standard - vertical, alternative layout }
Causative organism
Query

Causative organism

< Please choose >
causat_organ {Popup (Label Group)} **"causat_organ"**

specify other

causat_organ_oth {Textfield 30} **"causat_organ_oth"**

PVL

- positive (1 - 103717)
- negative (2 - 103718)

tested but unknown result (3 - 103719)

not tested (4 - 103720)

pvl {Vertical Radiobutton}

{ IASAlternateStandardGroup: .. Standard - vertical, alternative layout }

Blood culture

Blood culture

Query

positive (1 - 103717)

negative (2 - 103718)

not performed (3 - 103721)

unknown result (4 - 103722)

blood_culture {Vertical Radiobutton} **"Blood culture"**

{ IASHorizontalGroup: .... Standard - horizontal }

Time to positivity

Time to positivity

Query

hours

time_to_pos_h {Number 3,0, Missing Value}

minutes

time_to_pos_min {Number 3,0, Missing Value} **"Time to positivity"**

{ IASAlternateStandardGroup: .. Standard - vertical, alternative layout }

First line antibiotic treatment

First line antibiotic treatment

Query

Co-Amoxicillin

co_amoxicillin {Checkbox}

Penicillin

penicillin {Checkbox}

Flucloxacillin

flucloxacillin {Checkbox}

3G Cephoalosporin

cephoalosporin_3g {Checkbox}

4G Cephalosporin

cephoalosporin_4g {Checkbox}

Clindamycin

clindamycin {Checkbox}

Aminoglycosides

aminoglycosides {Checkbox}

Ciprofloxacin

ciprofloxacin {Checkbox}

Vancomycin

vancomycin {Checkbox}

Linezolid

linezolid {Checkbox}

Rifampicin

rifampicin {Checkbox}

Amoxicillin

amoxicillin {Checkbox}

other

other {Checkbox}

specify other

first_antibiotic_oth {Textfield 30} **"first_antibiotic_oth"**

First line Antibiotic treatment_notes

first_antibotic_note {Textarea 6,80}

{ IASAlternateStandardGroup: .. Standard - vertical, alternative layout }

Antibiotic treatment at discharge (oral)

Antibiotic treatment at discharge (oral)

Query

None

disch_antibiot_none {Checkbox}

Co-Amoxicillin

co_amoxicillin1 {Checkbox}

Penicillin

penicillin1 {Checkbox}

Flucloxacillin

flucloxacillin1 {Checkbox}

3G Cephoalosporin

cephoalosporin_3g1 {Checkbox}

4G Cephalosporin

cephoalosporin_4g1 {Checkbox}

Clindamycin

clindamycin1 {Checkbox}

Aminoglycosides

aminoglycosides1 {Checkbox}

Ciprofloxacin

ciprofloxacin1 {Checkbox}

Vancomycin

vancomycin1 {Checkbox}

Linezolid

linezolid1 {Checkbox}

Rifampicin

rifampicin1 {Checkbox}

Amoxicillin

amoxicillin1 {Checkbox}

other

other1 {Checkbox}

specify other

disch_antibiotic_oth {Textfield 30} **"disch_antibiotic_oth"**

Antibiotic treatment at discharge (oral)_notes

disch_antibotic_note {Textarea 6,80}

{ IASAlternateStandardGroup: .. Standard - vertical, alternative layout }
Antibiotics

Query

Days of intravenous administration

iv_days {Number 3,0}

Days of overall administration (iv and oral)

iv_oral_days {Number 3,0}

Is overall administration even conclusive (or is it not well documented?)

yes (1 - 101864) no (2 - 101747)

admin_conclusive {Horizontal Radiobutton}

Number of interventions (during hospital stay)

intervent_number {Number 2,0}

{ IASTableRepetitionGroup: .... Tabular repetition group , Initial count: 0, Max. count: unlimited, Show "Delete" button: yes, Display delete confirmation: no, Reverse repetitions: no, Initially ordered by: Displayed order }
detailled for each intervention

detailled for each intervention ***emnpukb08intervention***

{ IASHorizontalGroup: .... Standard - horizontal }
detailled for each intervention

No.
1

< Please choose >

intervent_details {Popup (Label Group)}

Query

More

{ IASAlternateStandardGroup: .. Standard - vertical, alternative layout }
Has patient been in aftercare at this hospital after discharged from hospital

Query

Has patient been in aftercare at this hospital after discharged from hospital

No visits

no_visits {Checkbox}

orthopedic follow-up for infection

orhto_fu {Checkbox}

readmission

readmission {Checkbox}

orthopedic outpatient visits

☐

ortho_outpat {Checkbox}

surgical intervention

☐

surgical_intervent {Checkbox}

specify

aftercare_detail {Textarea 6,80}

{ IASAlternateStandardGroup: .. Standard - vertical, alternative layout }

Comments

Comments

Query

optional

comments {Textarea 10,100}

---

emnpukb08bones.loc_of_inf_bone

1. Clavicula (1 - 101768)
2. Sternum (2 - 101769)
3. Humerus (3 - 101770)
4. Radius (4 - 101771)
5. Ulna (5 - 101772)
6. Pelvis (6 - 101773)
7. Femur (7 - 101774)
8. Tibia (8 - 101775)
9. Fibula (9 - 101776)
10. Talus (10 - 101777)
11. Calcaneus (11 - 101778)
12. Spine (12 - 101779)
13. other (99 - 101752)

causat_organ

2. Negative (1 - 103712)
3. St. Aureus (2 - 103713)
4. Strep pyogenes (3 - 103714)
5. Strep pneumoniae (4 - 103715)
6. Kingella kingae (5 - 103716)
7. other (99 - 101752)

emnpukb08intervention.intervent_details

1. Intraoperative microbiological sampling (1 - 103723)
2. lavage (2 - 103724)
3. curretage (3 - 103725)
4. installation of drainage (4 - 103726)
5. bone resection (5 - 103727)

6. unknown(missing data) (6 - 103728)

---

secuTrial® 5.5.0.12, 2019
