# Supplementary material for: Long-Term Outcomes of Acute Osteoarticular Infections in Children
Source: Front Pediatr. 2020 Nov 25;8:587740. doi: 10.3389/fped.2020.587740 (PMC7737431; doi:10.3389/fped.2020.587740)
Supplement: Supplementary file 2 [file Data_Sheet_2.PDF]

Interview mnpukb08interview

{ IASAlternateStandardGroup: .. Standard - vertical, alternative layout }
Type of Interview

Query

Type of Interview

Follow-up (1 - 101739) Phone (2 - 101740)

type_interview {Horizontal Radiobutton}

Date of interview

[ ] - [ ] - [ ] dd.mm.yyyy

date_interview {Date (dd.mm.yyyy), Date format: display}

{ IASAlternateStandardGroup: .. Standard - vertical, alternative layout }
Inclusion/Exclusion criteria

Query

Inclusion/Exclusion criteria

Inclusion criteria fulfillied

Inclusion criteria:
- Patients who were admitted to one of the participating centres between January 2005 and November 2014
- Patients with an International Classification of Diseases (ICD-10) discharge code of acute osteomyelitis (M86.00-M86.99) and/or septic arthritis (M00.00-M00.99)1

yes (1 - 101864) no (2 - 101747)

incl {Horizontal Radiobutton}

Exclusion criteria negated

Exclusion criteria:
1) Onset of symptoms > 2 weeks before admission
2) History of penetrating wound or prior surgery at affected limb
3) Incorrect ICD-10 coding
4) Chronic or severe underlying disease or treatment at time of AHOM/SA that possibly compromises the patient's immunologic response (e.g. cancer, immunodeficiency, immunosuppressive therapy)
5) Insufficient command of German language to understand patient information and informed consent

yes (1 - 101864) no (2 - 101747)

excl {Horizontal Radiobutton}

{ IASAlternateStandardGroup: .. Standard - vertical, alternative layout }
IC

Query

What type of consent form has been sent?

parents only (1 - 101741) child and parents (2 - 101742) adolescent (3 - 101743) adult (4 - 101744)

type_ic {Horizontal Radiobutton, Missing Value}

Has consent form been signed?

yes at visit (0 - 101745) yes at home (in case of phone interview) (1 - 101746) no (2 - 101747)

ic_signed {Horizontal Radiobutton}

Date of informed consent

[ ] - [ ] - [ ] dd.mm.yyyy

date_ic {Date (dd.mm.yyyy), Date format: display}

{ IASAlternateStandardGroup: .. Standard - vertical, alternative layout }
interview company

Query

Who did participant come with?

mother (1 - 101748) father (2 - 101749) both parents (3 - 101750) alone (4 - 101751) other (99 - 101752)

interview_accomp {Horizontal Radiobutton}

Interview was conducted with ...

participant (1 - 101753) legal guardian (2 - 101754) both (3 - 101755) other (99 - 101752)

interview_cond_with {Horizontal Radiobutton}

{ IASAlternateStandardGroup: .. Standard - vertical, alternative layout }
demographics

Query

Gender

male (1 - 101763) female (2 - 101764)

gender {Horizontal Radiobutton}

Age

.

age {Number 2,1}

{ IASAlternateStandardGroup: .. Standard - vertical, alternative layout }
Hospital

Query

Date of Admission

- - dd.mm.yyyy

date_admission {Date (dd.mm.yyyy), Date format: display}

Date of Discharge

- - dd.mm.yyyy

date_discharge {Date (dd.mm.yyyy), Date format: display}

What age was the patient at admission?

.

age_admission {Number 2,1}

{ IASAlternateStandardGroup: .. Standard - vertical, alternative layout }
icd

Query

Case type by ICD-Coding

OM (Osteomyelitis) (1 - 101765) SA (Septic Arthritis) (2 - 101766) OM + SA (3 - 101767)

icd_coding {Horizontal Radiobutton}

Participant's case type based on screening view

OM (Osteomyelitis) (1 - 101765) SA (Septic Arthritis) (2 - 101766) OM + SA (3 - 101767) OM with signs of joint involvement (4 - 105009) SA with signs of bone (5 - 105010) unconclusive (9 - 105011)

icd_coding_screen {Horizontal Radiobutton}

Number of locations

location_number {Number 2,0}

{ IASRepetitionGroup: .... Repetition group , Initial count: 1, Max. count: unlimited, Show "Delete" button: yes, Display delete confirmation: no, Reverse repetitions: no, Hide headline: no }
Affected body part

Affected body part

Infections 1 ***emnpukb08infections***

{ IASHorizontalGroup: .... Standard - horizontal }
infections

Query

Number of bones **Number of joints**

bones_count {Number 2,0, Missing Value}

joints_count {Number 2,0, Missing Value}

{ IASHorizontalGroup: .... Standard - horizontal }
bones spec

Query

Localisation of infection

< Please choose >

loc_of_inf_bone_1 {Popup (Label Group), Missing Value}

specify other

loc_inf_bone_oth_1 {Textfield 30}

Bone location

< Please choose >

loc_bone_1 {Popup (Label Group), Missing Value}

{ IASHorizontalGroup: .... Standard - horizontal }
bones spec 2

Query

Localisation of infection

< Please choose >

loc_of_inf_bone_2 {Popup (Label Group), Missing Value}

specify other

loc_inf_bone_oth_2 {Textfield 30}

Bone location

< Please choose >

loc_bone_2 {Popup (Label Group), Missing Value}

{ IASHorizontalGroup: .... Standard - horizontal }
bones spec 3

Query

Localisation of infection

< Please choose >

loc_of_inf_bone_3 {Popup (Label Group), Missing Value}

specify other

loc_inf_bone_oth_3 {Textfield 30}

Bone location

< Please choose >

loc_bone_3 {Popup (Label Group), Missing Value}

{ IASHorizontalGroup: .... Standard - horizontal }
joints spec

Query

Location of infection (joints)

< Please choose >

loc_of_inf_joint_1 {Popup (Label Group), Missing Value}

specify other

loc_inf_joint_oth_1 {Textfield 30}

{ IASHorizontalGroup: .... Standard - horizontal }
joints spec 2

Query

Location of infection (joints)

< Please choose >

loc_of_inf_joint_2 {Popup (Label Group), Missing Value}

specify other

loc_inf_joint_oth_2 {Textfield 30}

Delete
More

{ IASAlternateStandardGroup: .. Standard - vertical, alternative layout }
affected body part

Query

Side of infection

left (1 - 101860) right (2 - 101861)

infection_side {Horizontal Radiobutton}

Site of infection

upper extremity (1 - 101862) lower extremity (2 - 101863)

infection_site {Horizontal Radiobutton}

Localisation Comments

comments_loc {Textarea 6,80}

{ IASAlternateStandardGroup: .. Standard - vertical, alternative layout }
questions I

Query

If you hadn't received our letter, would you still have been able to recall that you have had that infection?

yes (1 - 101864) no (2 - 101747)

recall {Horizontal Radiobutton}

How do you describe your health regarding the previously infected body part?
4 is good and 1 is bad

< Please choose >

health {Popup (Label Group)}

If necessary, please specify

health_spec {Textarea 6,80}

Nowadays, do you experience pain in the previously affected body part?

never (0 - 101871) less than once month (1 - 101873) once a month (2 - 101876) once a week (4 - 101877) more than once a week (3 - 101878) daily (5 - 101879)

pain {Horizontal Radiobutton, Missing Value}

{ IASAlternateStandardGroup: .. Standard - vertical, alternative layout }
In what situations do you experience pain nowadays?

Query

In what situations do you experience pain nowadays?

pain at rest

pain_rest {Checkbox}

pain while standing

pain_standing {Checkbox}

pain while walking

pain_walking {Checkbox}

pain while climbing stairs

pain_stairs {Checkbox}

pain while doing sports/physical activity

pain_sports {Checkbox}

pain at night

pain_night {Checkbox}

don't remember

pain_dontremember {Checkbox}

{ IASAlternateStandardGroup: .. Standard - vertical, alternative layout }
pain II

Query

Do you take painkillers against it?

always (1 - 101885) sometimes (2 - 101886) never (0 - 101871)

painkillers {Horizontal Radiobutton}

If yes, have you ever have to call sick for work or school because of pain?

yes, once (0 - 101887) yes, several times (1 - 101888) no (2 - 101747) don't remember (9 - 101889) other (99 - 101752)

sick_pain {Horizontal Radiobutton}

specify other specify other

sick_pain_other {Textarea 3,80}

Notes on pain

comments_pain {Textarea 6,80}

{ IASAlternateStandardGroup: .. Standard - vertical, alternative layout }
recovery

Query

Do you remember when you felt as good as before the infection?

days after discharge (1 - 101890)
after 1 month (2 - 101891)
after 2-5 month (3 - 101892)
after 6 month (4 - 101893)
after 1 year (5 - 101894)
after many years (6 - 101895)
have never felt normal again (7 - 101896)
don't remember (9 - 101889)

felt_good_asbefore {Vertical Radiobutton}

{ IASAlternateStandardGroup: .. Standard - vertical, alternative layout }
Since discharge, have you been in medical care?

Query

Since discharge, have you been in medical care?

yes (1 - 101864) no (2 - 101747) don't remember (9 - 101889)

medicalcare_since {Horizontal Radiobutton}

{ IASAlternateStandardGroup: .. Standard - vertical, alternative layout }
Since discharge, have you been in medical care?

Query

trauma at affected limb

medc_trauma_limb {Checkbox}

other trauma

medc_trauma_other {Checkbox}

infection at affected limb

medc_infection_limb {Checkbox}

other infection

medc_infection_other {Checkbox}

neurologic

medc_neurologic {Checkbox}

orthopedic

medc_orthopedic {Checkbox}

respiratory/cardiac/gastrointestinal/urogenital

medc_respiratory_etc {Checkbox}

other

medc_other {Checkbox}

{ IASAlternateStandardGroup: .. Standard - vertical, alternative layout }
Notes on medical care

Query

Type of medical care

inpatient

inpatient_med_care {Checkbox}

outpatient

outpatient_med_care {Checkbox}

Notes on medical care

comments_med_care {Textarea 6,80}

{ IASAlternateStandardGroup: .. Standard - vertical, alternative layout }

Before admission, have you experienced any severe or chronic disease or trauma that has lead to medical care, outpatient or inpatient?

Query

Before admission, have you experienced any severe or chronic disease or trauma that has lead to medical care, outpatient or inpatient?

no sicknesses/trauma

before_no {Checkbox}

minor trauma

before_minor {Checkbox}

trauma fracture

before_fracture {Checkbox}

respiratory

before_respiratory {Checkbox}

cardiac

before_cardiac {Checkbox}

infection

before_infection {Checkbox}

neurological

before_neurological {Checkbox}

orthopedic

before_orthopedic {Checkbox}

other

before_other {Checkbox}

specify (how many times, what sicknesses)

before_comment {Textarea 6,80}

{ IASAlternateStandardGroup: .. Standard - vertical, alternative layout }

Regarding the previously affected body part: Have you ever been in aftercare after discharged from hospital

Query

Regarding the previously affected body part: Have you ever been in aftercare after discharged from hospital

no visits

aftercare_no {Checkbox}

orthopedic follow-up for infection

aftercare_infect {Checkbox}

readmission

aftercare_readmiss {Checkbox}

orthopedic outpatient visits

aftercare_outpat {Checkbox}

surgical intervention

aftercare_surgical {Checkbox}

physiotherapy

aftercare_physio {Checkbox}

{ IASAlternateStandardGroup: .. Standard - vertical, alternative layout }

specify (how many times, in the original hospital or in other hospital)

Query

specify (how many times, in the original hospital or in other hospital)

aftercare_specify {Textarea 6,80}

{ IASAlternateStandardGroup: .. Standard - vertical, alternative layout }

Are you still in aftercare today?

Query

Are you still in aftercare today?

no aftercare

aftercare2_no {Checkbox}

orthopedic visits

☐

aftercare2_orthop {Checkbox}

family doctor visits

☐

aftercare2_fam_doc {Checkbox}

physiotherapy

☐

aftercare2_physio {Checkbox}

{ IASAlternateStandardGroup: .. Standard - vertical, alternative layout }

specify today's aftercare

Query

specify today's aftercare

optional

aftercare2_specify {Textarea 6,80}

{ IASAlternateStandardGroup: .. Standard - vertical, alternative layout }

Sports

Query

Sports

How much sport do you do today (excluding school sports)?

- never (0 - 101871)
- less than once month (1 - 101873)
- once a month (2 - 101876)
- once a week (4 - 101877)
- 2-4 times a week (5 - 102259)
- almost daily (6 - 102260)

sport_amount {Vertical Radiobutton}

{ IASAlternateStandardGroup: .. Standard - vertical, alternative layout }

What kinds of sport

Query

What kinds of sport

ball sports

☐

ball_sports {Checkbox}

endurance_sports

☐

endurance_sports {Checkbox}

walking/hiking

walking_sports {Checkbox}

{ IASAlternateStandardGroup: .. Standard - vertical, alternative layout }
specify the kind of sport you do

Query

specify the kind of sport you do

sport_specify {Textarea 6,80}

How competitive do you do sports?

leisure (1 - 102261) hig-performance (2 - 102262)

sport_competitive {Horizontal Radiobutton}

{ IASAlternateStandardGroup: .. Standard - vertical, alternative layout }
The year before admission, how much sport did you do on average (excluding school)?

Query

The year before admission, how much sport did you do on average (excluding school)?

never (0 - 101871)
less than once month (1 - 101873)
once a month (2 - 101876)
once a week (4 - 101877)
2-4 times a week (5 - 102259)
almost daily (6 - 102260)

sport2_amount {Vertical Radiobutton}

{ IASAlternateStandardGroup: .. Standard - vertical, alternative layout }
What kinds of sport

Query

What kinds of sport

ball sports

ball_sports2 {Checkbox}

endurance_sports

endurance_sports2 {Checkbox}

walking/hiking

walking_sports2 {Checkbox}

{ IASAlternateStandardGroup: .. Standard - vertical, alternative layout }
specify the kind of sport you did

Query

specify the kind of sport you did

sport2_specify {Textarea 6,80}

How competitive did you do sports?

- leisure (1 - 102261)
- hig-performance (2 - 102262)

sport2_competitive {Horizontal Radiobutton}

{ IASAlternateStandardGroup: .. Standard - vertical, alternative layout }

Until one year after discharge, how much sport did you do on average (excluding school sports)?

Query

Until one year after discharge, how much sport did you do on average (excluding school sports)?

- never (0 - 101871)
- less than once month (1 - 101873)
- once a month (2 - 101876)
- once a week (4 - 101877)
- 2-4 times a week (5 - 102259)
- almost daily (6 - 102260)

sport3_amount {Vertical Radiobutton}

{ IASAlternateStandardGroup: .. Standard - vertical, alternative layout }

What kinds of sport

Query

What kinds of sport

ball sports

ball_sports3 {Checkbox}

endurance_sports

endurance_sports3 {Checkbox}

walking/hiking

walking_sports3 {Checkbox}

{ IASAlternateStandardGroup: .. Standard - vertical, alternative layout }

specify the kind of sport you did

Query

specify the kind of sport you did

sport3_specify {Textarea 6,80}

How competitive did you do sports?

leisure (1 - 102261) hig-performance (2 - 102262)

sport3_competitive {Horizontal Radiobutton}

{ IASAlternateStandardGroup: .. Standard - vertical, alternative layout }

Comments

Comments

Query

optional

comments {Textarea 10,100}

---

emnpukb08infections.loc_of_inf_bone_1

1. Clavicula (1 - 101768)
2. Sternum (2 - 101769)
3. Humerus (3 - 101770)
4. Radius (4 - 101771)
5. Ulna (5 - 101772)
6. Pelvis (6 - 101773)
7. Femur (7 - 101774)
8. Tibia (8 - 101775)
9. Fibula (9 - 101776)
10. Talus (10 - 101777)
11. Calcaneus (11 - 101778)
12. Spine (12 - 101779)
13. other (99 - 101752)

emnpukb08infections.loc_bone_1

1. proximal epiphysis (1 - 101780)
2. proximal metaphysis (2 - 101781)
3. proximal diaphysis (3 - 101782)
4. central diaphysis (4 - 101783)
5. distal diaphysis (5 - 101784)
6. distal metaphysis (6 - 101785)
7. distal episphysis (7 - 101786)
8. not further specified (9 - 101787)

emnpukb08infections.loc_of_inf_bone_2

1. Clavicula (1 - 101768)
2. Sternum (2 - 101769)
3. Humerus (3 - 101770)
4. Radius (4 - 101771)
5. Ulna (5 - 101772)
6. Pelvis (6 - 101773)
7. Femur (7 - 101774)
8. Tibia (8 - 101775)
9. Fibula (9 - 101776)
10. Talus (10 - 101777)
11. Calcaneus (11 - 101778)
12. Spine (12 - 101779)
13. other (99 - 101752)

emnpukb08infections.loc_bone_2

1. proximal epiphysis (1 - 101780)
2. proximal metaphysis (2 - 101781)
3. proximal diaphysis (3 - 101782)
4. central diaphysis (4 - 101783)
5. distal diaphysis (5 - 101784)
6. distal metaphysis (6 - 101785)
7. distal episphysis (7 - 101786)
8. not further specified (9 - 101787)

emnpukb08infections.loc_of_inf_bone_3

1. Clavicula (1 - 101768)

	2. Sternum (2 - 101769)<br>3. Humerus (3 - 101770)<br>4. Radius (4 - 101771)<br>5. Ulna (5 - 101772)<br>6. Pelvis (6 - 101773)<br>7. Femur (7 - 101774)<br>8. Tibia (8 - 101775)<br>9. Fibula (9 - 101776)<br>10. Talus (10 - 101777)<br>11. Calcaneus (11 - 101778)<br>12. Spine (12 - 101779)<br>13. other (99 - 101752)
emnpukb08infections.loc_bone_3	1. proximal epiphysis (1 - 101780)<br>2. proximal metaphysis (2 - 101781)<br>3. proximal diaphysis (3 - 101782)<br>4. central diaphysis (4 - 101783)<br>5. distal diaphysis (5 - 101784)<br>6. distal metaphysis (6 - 101785)<br>7. distal episphysis (7 - 101786)<br>8. not further specified (9 - 101787)
emnpukb08infections.loc_of_inf_joint_1	1. Shoulder (1 - 101788)<br>2. Elbow (2 - 101789)<br>3. Wrist (3 - 101790)<br>4. SIJ (4 - 101791)<br>5. Hip (5 - 101792)<br>6. Knee (6 - 101793)<br>7. Talocrural joint (7 - 101794)<br>8. other (99 - 101752)
emnpukb08infections.loc_of_inf_joint_2	1. Shoulder (1 - 101788)<br>2. Elbow (2 - 101789)<br>3. Wrist (3 - 101790)<br>4. SIJ (4 - 101791)<br>5. Hip (5 - 101792)<br>6. Knee (6 - 101793)<br>7. Talocrural joint (7 - 101794)<br>8. other (99 - 101752)
health	1. 1 (1 - 101865)<br>2. 2 (2 - 101866)<br>3. 3 (3 - 101867)<br>4. 4 (4 - 101868)<br>5. 5 (5 - 103699)

secuTrial® 5.5.0.12, 2019
