# Supplementary material for: Long-Term Outcomes of Acute Osteoarticular Infections in Children
Source: Front Pediatr. 2020 Nov 25;8:587740. doi: 10.3389/fped.2020.587740 (PMC7737431; doi:10.3389/fped.2020.587740)
Supplement: Supplementary file 3 [file Data_Sheet_3.PDF]

Physical activity mnpukb08phys_activity

{ IASAlternateStandardGroup: .. Standard - vertical, alternative layout }
date

Query

Date of interview

[ ] - [ ] - [ ] dd.mm.yyyy

date_interview {Date (dd.mm.yyyy), Date format: display}

{ IASAlternateStandardGroup: .. Standard - vertical, alternative layout }
LOWER EXTREMITY FUNCTION

Query

LOWER EXTREMITY FUNCTION

Tested

( ) yes (1 - 101864) ( ) no (2 - 101747)

lower_extremity {Horizontal Radiobutton}

{ IASAlternateStandardGroup: .. Standard - vertical, alternative layout }
Can you run short distances?

Query

Can you run short distances?

( ) Yes easily (1 - 102267) ( ) Yes, but a little hard (2 - 102268) ( ) Yes, but very hard (3 - 102269) ( ) No (4 - 102270)

distance_short {Horizontal Radiobutton}

pain in affected body part

[ ]

pain_affect_short {Checkbox}

pain at other body part

[ ]

pain_other_short {Checkbox}

general health

[ ]

health_short {Checkbox}

social reasons

[ ]

social_short {Checkbox}

dislike

[ ]

dislike_short {Checkbox}

other

[ ]

other_short {Checkbox}

specify

[ ]

other_spec_short {Textfield 60}

{ IASAlternateStandardGroup: .. Standard - vertical, alternative layout }
Can you run long distances?

Query

Can you run long distances?

Yes easily (1 - 102267) Yes, but a little hard (2 - 102268) Yes, but very hard (3 - 102269) No (4 - 102270)

distance_long {Horizontal Radiobutton}

participant too young to assess this activity

too_young_long {Checkbox}

pain in affected body part

pain_affect_long {Checkbox}

pain at other body part

pain_other_long {Checkbox}

general health

health_long {Checkbox}

social reasons

social_long {Checkbox}

dislike

dislike_long {Checkbox}

other

other_long {Checkbox}

specify

other_spec_long {Textfield 60}

{ IASAlternateStandardGroup: .. Standard - vertical, alternative layout }

Can you walk more than 100m?

Can you walk more than 100m?

Query

Yes easily (1 - 102267) Yes, but a little hard (2 - 102268) Yes, but very hard (3 - 102269) No (4 - 102270)

distance_100m {Horizontal Radiobutton}

pain in affected body part

pain_affect_100m {Checkbox}

pain at other body part

pain_other_100m {Checkbox}

general health

health_100m {Checkbox}

social reasons

☐

social_100m {Checkbox}

dislike

☐

dislike_100m {Checkbox}

other

☐

other_100m {Checkbox}

specify

[ ]

other_spec_100m {Textfield 60}

{ IASAlternateStandardGroup: .. Standard - vertical, alternative layout }

Can you walk more than 1000m?

Can you walk more than 1000m?

Query

○ Yes easily (1 - 102267) ○ Yes, but a little hard (2 - 102268) ○ Yes, but very hard (3 - 102269) ○ No (4 - 102270)

distance_1000m {Horizontal Radiobutton}

pain in affected body part

☐

pain_affect_1000m {Checkbox}

pain at other body part

☐

pain_other_1000m {Checkbox}

general health

☐

health_1000m {Checkbox}

social reasons

☐

social_1000m {Checkbox}

dislike

☐

dislike_1000m {Checkbox}

other

☐

other_1000m {Checkbox}

specify

[ ]

other_spec_1000m {Textfield 60}

{ IASAlternateStandardGroup: .. Standard - vertical, alternative layout }
What is the longest distance you can walk

What is the longest distance you can walk

Query

longest_dist_walk {Textarea 6,80} **"What is the longest distance you can walk"**

{ IASAlternateStandardGroup: .. Standard - vertical, alternative layout }
Can you climb stairs?

Can you climb stairs?

Query

Yes easily (1 - 102267) Yes, but a little hard (2 - 102268) Yes, but very hard (3 - 102269) No (4 - 102270)

stair_climb {Horizontal Radiobutton}

pain in affected body part

pain_affect_stair {Checkbox}

pain at other body part

pain_otherstair {Checkbox}

general health

health_stair {Checkbox}

social reasons

social_stair {Checkbox}

dislike

dislike_stair {Checkbox}

other

other_stair {Checkbox}

specify

other_spec_stair {Textfield 60}

{ IASAlternateStandardGroup: .. Standard - vertical, alternative layout }
Can you walk uphill?

Can you walk uphill?

Query

Yes easily (1 - 102267) Yes, but a little hard (2 - 102268) Yes, but very hard (3 - 102269) No (4 - 102270)

uphill_walk {Horizontal Radiobutton}

pain in affected body part

pain_affect_uphill {Checkbox}

pain at other body part

pain_other_uphill {Checkbox}

general health

health_uphill {Checkbox}

social reasons

social_uphill {Checkbox}

dislike

dislike_uphill {Checkbox}

other

other_uphill {Checkbox}

specify

other_spec_uphill {Textfield 60}

{ IASAlternateStandardGroup: .. Standard - vertical, alternative layout }

Can you walk downhill?

Query

Can you walk downhill?

Yes easily (1 - 102267) Yes, but a little hard (2 - 102268) Yes, but very hard (3 - 102269) No (4 - 102270)

downhill_walk {Horizontal Radiobutton}

pain in affected body part

pain_affect_downhill {Checkbox}

pain at other body part

pain_other_downhill {Checkbox}

general health

health_downhill {Checkbox}

social reasons

social_downhill {Checkbox}

dislike

dislike_downhill {Checkbox}

other

☐

other_downhill {Checkbox}

specify

other_spec_downhill {Textfield 60}

{ IASAlternateStandardGroup: .. Standard - vertical, alternative layout }

Can you participate in ball sports (basketball, soccer, tennis, hockey)?

Can you participate in ball sports (basketball, soccer, tennis, hockey)?

Query

○ Yes easily (1 - 102267) ○ Yes, but a little hard (2 - 102268) ○ Yes, but very hard (3 - 102269) ○ No (4 - 102270)

sports {Horizontal Radiobutton}

pain in affected body part

☐

pain_affect_sports {Checkbox}

pain at other body part

☐

pain_other_sports {Checkbox}

general health

☐

health_sports {Checkbox}

social reasons

☐

social_sports {Checkbox}

dislike

☐

dislike_sports {Checkbox}

other

☐

other_sports {Checkbox}

specify

other_spec_sports {Textfield 60}

{ IASAlternateStandardGroup: .. Standard - vertical, alternative layout }

Can you kneel down?

Can you kneel down?

Query

○ Yes easily (1 - 102267) ○ Yes, but a little hard (2 - 102268) ○ Yes, but very hard (3 - 102269) ○ No (4 - 102270)

kneel {Horizontal Radiobutton}

pain in affected body part

☐

pain_affect_kneel {Checkbox}

pain at other body part

☐

pain_other_kneel {Checkbox}

general health

☐

health_kneel {Checkbox}

social reasons

☐

social_kneel {Checkbox}

dislike

☐

dislike_kneel {Checkbox}

other

☐

other_kneel {Checkbox}

specify

other_spec_kneel {Textfield 60}

{ IASAlternateStandardGroup: .. Standard - vertical, alternative layout }

Can you sit on a sofa or in a car for an hour?

Query

Can you sit on a sofa or in a car for an hour?

○ Yes easily (1 - 102267) ○ Yes, but a little hard (2 - 102268) ○ Yes, but very hard (3 - 102269) ○ No (4 - 102270)

sit {Horizontal Radiobutton}

pain in affected body part

☐

pain_affect_sit {Checkbox}

pain at other body part

☐

pain_other_sit {Checkbox}

general health

☐

health_sit {Checkbox}

social reasons

☐

social_sit {Checkbox}

dislike

☐

dislike_sit {Checkbox}

other

other_sit {Checkbox}

specify

other_spec_sit {Textfield 60}

{ IASAlternateStandardGroup: .. Standard - vertical, alternative layout }

Notes on physical activities of lower limb

Query

Notes on physical activities of lower limb

comments_lower_limb {Textarea 6,80}

{ IASAlternateStandardGroup: .. Standard - vertical, alternative layout }

UPPER EXTREMITY FUNCTION

Query

UPPER EXTREMITY FUNCTION

Tested

yes (1 - 101864) no (2 - 101747)

upper_extremity {Horizontal Radiobutton}

{ IASAlternateStandardGroup: .. Standard - vertical, alternative layout }

Can you use the affected limb to do light work e.g. getting dressed, showering?

Query

Can you use the affected limb to do light work e.g. getting dressed, showering?

Yes easily (1 - 102267) Yes, but a little hard (2 - 102268) Yes, but very hard (3 - 102269) No (4 - 102270)

lightwork {Horizontal Radiobutton}

pain in affected body part

pain_affect_lightwor {Checkbox}

pain at other body part

pain_other_lightwork {Checkbox}

general health

health_lightwork {Checkbox}

social reasons

social_lightwork {Checkbox}

dislike

dislike_lightwork {Checkbox}

other

other_lightwork {Checkbox}

specify

other_spec_lightwork {Textfield 60}

{ IASAlternateStandardGroup: .. Standard - vertical, alternative layout }

Can you use the affected limb to do heavy work e.g. lifting a suitcase?

Can you use the affected limb to do heavy work e.g. lifting a suitcase?

Query

Yes easily (1 - 102267) Yes, but a little hard (2 - 102268) Yes, but very hard (3 - 102269) No (4 - 102270)

heavywork {Horizontal Radiobutton}

participant too young to assess this activity

too_young_heavywork {Checkbox}

pain in affected body part

pain_affect_heavywor {Checkbox}

pain at other body part

pain_other_heavywork {Checkbox}

general health

health_heavywork {Checkbox}

social reasons

social_heavywork {Checkbox}

dislike

dislike_heavywork {Checkbox}

other

other_heavywork {Checkbox}

specify

other_spec_heavywork {Textfield 60}

{ IASAlternateStandardGroup: .. Standard - vertical, alternative layout }

Can you use the affected limb for tools e.g. screwdriver?

Can you use the affected limb for tools e.g. screwdriver?

Query

Yes easily (1 - 102267) Yes, but a little hard (2 - 102268) Yes, but very hard (3 - 102269) No (4 - 102270)

tools {Horizontal Radiobutton}

pain in affected body part

pain_affect_tools {Checkbox}

pain at other body part

pain_other_tools {Checkbox}

general health

health_tools {Checkbox}

social reasons

social_tools {Checkbox}

dislike

dislike_tools {Checkbox}

other

other_tools {Checkbox}

specify

other_spec_tools {Textfield 60}

{ IASAlternateStandardGroup: .. Standard - vertical, alternative layout }

Can you use the affected limb to put weight on e.g. plan position?

Can you use the affected limb to put weight on e.g. plan position?

Query

Yes easily (1 - 102267) Yes, but a little hard (2 - 102268) Yes, but very hard (3 - 102269) No (4 - 102270)

weight {Horizontal Radiobutton}

pain in affected body part

pain_affect_weight {Checkbox}

pain at other body part

pain_other_weight {Checkbox}

general health

health_weight {Checkbox}

social reasons

social_weight {Checkbox}

dislike

dislike_weight {Checkbox}

other

☐

other_weight {Checkbox}

specify

other_spec_weight {Textfield 60}

{ IASAlternateStandardGroup: .. Standard - vertical, alternative layout }
Can you use the affected limb to work overhead e.g. grabbing something from a high shelf?

Query

Can you use the affected limb to work overhead e.g. grabbing something from a high shelf?

○ Yes easily (1 - 102267) ○ Yes, but a little hard (2 - 102268) ○ Yes, but very hard (3 - 102269) ○ No (4 - 102270)

overhead {Horizontal Radiobutton}

pain in affected body part

☐

pain_affect_overhead {Checkbox}

pain at other body part

☐

pain_other_overhead {Checkbox}

general health

☐

health_overhead {Checkbox}

social reasons

☐

social_overhead {Checkbox}

dislike

☐

dislike_overhead {Checkbox}

other

☐

other_overhead {Checkbox}

specify

other_spec_overhead {Textfield 60}

{ IASAlternateStandardGroup: .. Standard - vertical, alternative layout }
Notes on physical activities of upper limb

Query

Notes on physical activities of upper limb

comments_upper_limb {Textarea 6,80}

{ IASHeadline: .... Headline }

Current state of health

{ IASAlternateStandardGroup: .. Standard - vertical, alternative layout }
current state of health

Query

Now, after all these questions: How do you describe your health regarding the previously infected body part?
If 5 is good and 1 is bad

< Please choose >

health_score {Popup (Label Group)}

If necessary, please specify

comments_health {Textarea 6,80}

{ IASAlternateStandardGroup: .. Standard - vertical, alternative layout }
doc current state of health

Query

Doctor's perspective: How would you describe the health of the participant regarding the previously infected body part?
If 5 is good and 1 is bad

< Please choose >

health_score_doc {Popup (Label Group)}

If necessary, please specify

comments_health_doc {Textarea 6,80}

{ IASAlternateStandardGroup: .. Standard - vertical, alternative layout }
Comments

Comments

Query

optional

comments {Textarea 10,100}

secuTrial® 5.5.0.12, 2019
