# Supplementary material for: Long-Term Outcomes of Acute Osteoarticular Infections in Children
Source: Front Pediatr. 2020 Nov 25;8:587740. doi: 10.3389/fped.2020.587740 (PMC7737431; doi:10.3389/fped.2020.587740)
Supplement: Supplementary file 4 [file Data_Sheet_4.PDF]

Examination mnpukb08examination

{ IASAlternateStandardGroup: .. Standard - vertical, alternative layout }
date

Query

Date of examination

[ ] - [ ] - [ ] dd.mm.yyyy

date_examination {Date (dd.mm.yyyy), Date format: display}

{ IASAlternateStandardGroup: .. Standard - vertical, alternative layout }
Was examination performed

Query

Was examination performed

- ( ) examination performed (1 - 102583)
- ( ) no examination (phone interview) (2 - 102584)
- ( ) no examination (other reason) (3 - 102585)

performed {Vertical Radiobutton} **"was examination performed"**

Notes on examination e.g. why was examination not performed?
optional

[ ]

examination_com {Textarea 3,80}

FOTO DOCUMENTATION

( ) allowed (1 - 102586) ( ) not allowd (0 - 102587)

foto_doc {Horizontal Radiobutton}

VIDEO DOCUMENTATION

( ) allowed (1 - 102586) ( ) not allowd (0 - 102587)

video_doc {Horizontal Radiobutton}

WAS VALIDATION PERFORMED?

- ( ) Yes all of it with Andi Krieg (1 - 102588)
- ( ) Yes part of it with Andi Krieg (2 - 102589)
- ( ) Yes all of it with other orthopaedist (3 - 102590)
- ( ) Yes part of it with other orthopaedist (4 - 102591)
- ( ) No not yet (5 - 102592)
- ( ) No not necessary (no questions) (6 - 102593)

validation_performed {Vertical Radiobutton}

OTHER ORTHOPAEDIST'S NAME

[ ]

orthop_name {Textfield 60}

Date of validation

[ ] - [ ] - [ ] dd.mm.yyyy

date_validation {Date (dd.mm.yyyy), Date format: display}

{ IASHeadline: .... Headline }

LOWER EXTREMITY

{ IASAlternateStandardGroup: .. Standard - vertical, alternative layout }
GAIT, STANDING and ALIGNEMENT

Query

GAIT, STANDING and ALIGNEMENT tested

yes (1 - 101864) no (2 - 101747)

gait_tested {Horizontal Radiobutton}

Height

. cm

height {Number 3,1}

{ IASAlternateStandardGroup: .. Standard - vertical, alternative layout }
INSPECTION OF LEG AXIS_standing

Query

INSPECTION OF LEG AXIS_standing

symmetric (1 - 102594) asymmetric (2 - 102595)

leg_axis_standing {Horizontal Radiobutton}

1 = not further described
2 = more genu varum in affected limb
3 = more genu varum in unaffected limb
4 = more genu valgum in affected limb
5 =more genu valgum in unaffected limb
6 = more genu flexum in affected limb
7 = more genu flexum in unaffected limb
8 = more genu recurvatum in affected limb
9 = more genu recurvatum in unaffected limb
other

< Please choose >

leg_axis_stand_det {Popup (Label Group)} "leg_axis_stand_details"

specify

leg_axis_stand_oth {Textfield 80} "leg_axis_stand_oth"

{ IASAlternateStandardGroup: .. Standard - vertical, alternative layout }
INSPECTION OF VARUS/VALGUS DEFORMITY OF THE TALOCRURAL JOINT

Query

INSPECTION OF VARUS/VALGUS DEFORMITY OF THE TALOCRURAL JOINT

symmetric (1 - 102594) asymmetric (2 - 102595)

talocrural {Horizontal Radiobutton}

1 = not further described
2 = more valgus in affected leg 3 = more valgus in unaffected leg
4 = more varus in affected leg
5 = more varus in unaffected leg
other

< Please choose >

talocrural_det {Popup (Label Group)} "TALOCRURAL_details"

specify

talocrural_oth {Textfield 80} "TALOCRURAL_oth"

{ IASAlternateStandardGroup: .. Standard - vertical, alternative layout }
INSPECTION OF TALOCRURAL AXIS

Query

INSPECTION OF TALOCRURAL AXIS

physiological varization (1 - 103704) no varization (0 - 103705)

talocrural_axis {Horizontal Radiobutton}

{ IASAlternateStandardGroup: .. Standard - vertical, alternative layout }
PELVIC OBLIQUITY

Query

PELVIC OBLIQUITY
number of centimeters required to reestablish symmetry

< Please choose >

pelvic_obl {Popup (Label Group)}

{ IASAlternateStandardGroup: .. Standard - vertical, alternative layout }
INSPECTION OF LEG AXIS_walking

Query

INSPECTION OF LEG AXIS_walking

symmetric (1 - 102594) asymmetric (2 - 102595)

leg_axis_walk {Horizontal Radiobutton}

1= not further described
2 = more internal rotation in affected leg
3 = more internal rotation in unaffected leg
4 = more external rotation in affected leg
5 = more external rotation in unaffected leg
other

< Please choose >

leg_axis_walk_det {Popup (Label Group)} "leg_axis_walk_details"

specify

leg_axis_walk_oth {Textfield 80} "leg_axis_walk_oth"

{ IASAlternateStandardGroup: .. Standard - vertical, alternative layout }
Inspection of gait: participant walks through examination room

Query

Inspection of gait: participant walks through examination room

symmetric (1 - 102594) limping (2 - 104224)

gait_walk {Horizontal Radiobutton}

{ IASAlternateStandardGroup: .. Standard - vertical, alternative layout }
Inspection of femoral torsion (prone position):

Query

Inspection of femoral torsion (prone position)

symmetric (1 - 102594) asymmetric (2 - 102595)

fem_tor {Horizontal Radiobutton}

1 = not further described
2 = more antetorsion in affected limb
3 = more antetorsion in unaffected limb
other

< Please choose >

fem_tor_det {Popup (Label Group)} **"femoral torsion prone detail"**

specify

fem_tor_oth {Textfield 80} **"fem_tor_prone_oth"**

{ **IASAlternateStandardGroup: .. Standard - vertical, alternative layout** }

Inspection of tibial torsion (prone position, 90° flexion in knee)

Query

Inspection of tibial torsion (prone position, 90° flexion in knee)

symmetric (1 - 102594) asymmetric (2 - 102595)

tib_tor {Horizontal Radiobutton}

1 = not further described
2 = more internal rotation in affected tibia
3 = more internal rotation in unaffected tibia
4 = more external rotation in affected tibia
5 = more external rotation in unaffected tibia
other

< Please choose >

tib_tor_det {Popup (Label Group)} **"femoral torsion prone detail"**

specify

tib_tor_oth {Textfield 80} **"tib_tor_other"**

{ **IASAlternateStandardGroup: .. Standard - vertical, alternative layout** }

Comments

Query

Notes (gait)

optional

notes_gait {Textarea 10,100} **"Notes (gait)"**

{ **IASHeadline: .... Headline** }

SPECIFIC EXAMINATION: joints tested according to study protocol

{ **IASAlternateStandardGroup: .. Standard - vertical, alternative layout** }

HIP

Query

HIP

Tested?

yes (1 - 101864) no (2 - 101747)

tested_hip {Horizontal Radiobutton}

Internal rotation (in prone position and supine position)
1 = symmetric
2 = deficit in affected
3 = deficit in unaffected

1 (1 - 101865) 2 (2 - 101866) 3 (3 - 101867)

hip_int_rot {Horizontal Radiobutton}

Extent of internal rotation deficit (degrees)

°

hip_int_rot_deg {Number 2,0}

External rotation (in prone position and supine position)
1 = symmetric
2 = deficit in affected
3 = deficit in unaffected

1 (1 - 101865) 2 (2 - 101866) 3 (3 - 101867)

hip_ext_rot {Horizontal Radiobutton}

Extent of external rotation deficit (degrees)

°

hip_ext_rot_deg {Number 2,0}

Was there any pain while examining rotation of the legs
1 = In affected limb
2 = in other limb
3 = both
4 = No

1 (1 - 101865) 2 (2 - 101866) 3 (3 - 101867) 4 (4 - 101868)

hip_rot_pain {Horizontal Radiobutton}

{ **IASAlternateStandardGroup: .. Standard - vertical, alternative layout** }
HIP abduc

Query

Abduction in both legs (supine position)
1 = symmetric
2 = deficit in affected
3 = deficit in unaffected

1 (1 - 101865) 2 (2 - 101866) 3 (3 - 101867)

hip_abduc {Horizontal Radiobutton}

Extent of abduction deficit

abduc_def {Number 2,0}

Was there any pain while examining abduction of the legs
1 = In affected limb
2 = in other limb
3 = both
4 = No

1 (1 - 101865) 2 (2 - 101866) 3 (3 - 101867) 4 (4 - 101868)

hip_abduc_pain {Horizontal Radiobutton}

{ IASAlternateStandardGroup: .. Standard - vertical, alternative layout }
HIP flex and ext

Query

Thomas test for both legs (supine position)
1 = no deficit
2 = flexion contracture of affected hip
3 = flexion contracture of unaffected hip
4 = flexion contracture in both hips

1 (1 - 101865) 2 (2 - 101866) 3 (3 - 101867) 4 (4 - 101868)

hips_thomas_both {Horizontal Radiobutton}

Flexion in both hips (supine position)
1 = symmetric
2 = deficit in affected
3 = deficit in unaffected

1 (1 - 101865) 2 (2 - 101866) 3 (3 - 101867)

hip_flex_both {Horizontal Radiobutton}

Flexion in both hips (supine position)

hips_flex_def {Number 3,0}

Extension in both hips (prone position)
1 = symmetric
2 = deficit in affected
3 = deficit in unaffected

1 (1 - 101865) 2 (2 - 101866) 3 (3 - 101867)

hip_ext_both {Horizontal Radiobutton}

Extent of extension deficit (degree)

hips_ext_def {Number 2,0}

Drehmann-sign

no drehmann sign (0 - 105012) positive drehmann sign (1 - 105013)

hip_drehmann_sign {Horizontal Radiobutton}

Was there any pain while examining flexion and extension of the legs
1 = In affected limb
2 = in other limb
3 = both
4 = No

1 (1 - 101865) 2 (2 - 101866) 3 (3 - 101867) 4 (4 - 101868)

hip_flex_ext_pain {Horizontal Radiobutton}

Notes hip examination
optional

notes_hip {Textarea 10,100}

{ IASAlternateStandardGroup: .. Standard - vertical, alternative layout }

KNEE

KNEE

Query

Tested?

yes (1 - 101864) no (2 - 101747)

tested_knee {Horizontal Radiobutton}

Flexion (supine position)

1 = symmetric
2 = deficit in affected
3 = deficit in unaffected

1 (1 - 101865) 2 (2 - 101866) 3 (3 - 101867)

knee_flex {Horizontal Radiobutton}

Extent of flexion deficit (degrees)

°

knee_flex_deficit {Number 3,0}

Extension (supine position)

1 = symmetric
2 = deficit in affected
3 = deficit in unaffected

1 (1 - 101865) 2 (2 - 101866) 3 (3 - 101867)

knee_ext {Horizontal Radiobutton}

Extent of extension deficit

°

knee_ext_def {Number 2,0}

Was there any pain while examining flexion and extension of the knees

1 = In affected limb
2 = in other limb
3 = both
4 = No

1 (1 - 101865) 2 (2 - 101866) 3 (3 - 101867) 4 (4 - 101868)

knee_pain {Horizontal Radiobutton}

Notes knee examination

optional

notes_knee {Textarea 10,100}

{ IASAlternateStandardGroup: .. Standard - vertical, alternative layout }

TALOCRURAL JOINT

Query

TALOCRURAL JOINT

Tested?

yes (1 - 101864) no (2 - 101747)

tested_talocrural {Horizontal Radiobutton}

Plantar flexion

1 = symmetric
2 = deficit in affected
3 = deficit in unaffected

1 (1 - 101865) 2 (2 - 101866) 3 (3 - 101867)

talocr_flex {Horizontal Radiobutton}

Extent of flexion deficit

°

talocr_flex_def {Number 2,0}

Dorsal extension

1 = symmetric
2 = deficit in affected
3 = deficit in unaffected

1 (1 - 101865) 2 (2 - 101866) 3 (3 - 101867)

talocr_ext {Horizontal Radiobutton}

Extent of extension deficit

°

talocr_ext_def {Number 2,0}

Was there any pain while examining flexion and extension of the feet

1 = In affected limb
2 = in other limb
3 = both
4 = No

1 (1 - 101865) 2 (2 - 101866) 3 (3 - 101867) 4 (4 - 101868)

talocr_flex_ext_pain {Horizontal Radiobutton}

Pronation in both ankles

1 = symmetric
2 = deficit in affected
3 = deficit in unaffected

1 (1 - 101865) 2 (2 - 101866) 3 (3 - 101867)

talocr_pron {Horizontal Radiobutton}

Supination in both ankles

1 = symmetric
2 = deficit in affected
3 = deficit in unaffected

1 (1 - 101865) 2 (2 - 101866) 3 (3 - 101867)

talocr_supin {Horizontal Radiobutton}

Was there any pain while examining pro- and supination in ankle

1 = In affected limb
2 = in other limb
3 = both
4 = No

1 (1 - 101865) 2 (2 - 101866) 3 (3 - 101867) 4 (4 - 101868)

talocr_pro_n_pain {Horizontal Radiobutton}

Notes talocrural examination

optional

notes_talocr {Textarea 10,100}

{ IASHeadline: .... Headline }

UPPER EXTREMITY

{ IASAlternateStandardGroup: .. Standard - vertical, alternative layout }

LENGTH AND ALIGNEMENT UPPER EXTR

Query

LENGTH AND ALIGNEMENT tested

yes (1 - 101864) no (2 - 101747)

upper_tested {Horizontal Radiobutton}

Length deficit

Assessment of arm length: placing palms on each other: lenght deficit when comparing fingers of both hands?

. cm

length_arm_def {Number 2,1}

Inspection of arm axis

1 = Symmetric arm axis
2= more varus in affected limb
3 = more varus in unaffected limb
4 = more valgus in affected limb
5 = more valgus in unaffected limb

1 (1 - 101865) 2 (2 - 101866) 3 (3 - 101867) 4 (4 - 101868) 5 (5 - 103699)

arm_axis {Horizontal Radiobutton}

{ IASAlternateStandardGroup: .. Standard - vertical, alternative layout }

SHOULDER

SHOULDER

Query

Tested?

yes (1 - 101864) no (2 - 101747)

tested_should {Horizontal Radiobutton}

Forward flexion

1 = symmetric
2 = deficit in affected
3 = deficit in unaffected

1 (1 - 101865) 2 (2 - 101866) 3 (3 - 101867)

should_flex {Horizontal Radiobutton}

Extent of forward flexion deficit

°

should_flex_def {Number 3,0}

Extension

1 = symmetric
2 = deficit in affected
3 = deficit in unaffected

1 (1 - 101865) 2 (2 - 101866) 3 (3 - 101867)

should_ext {Horizontal Radiobutton}

Extent of extension deficit

°

should_ext_def {Number 2,0}

Was there any pain while examining flexion and extension of the shoulders

1 = In affected limb
2 = in other limb
3 = both
4 = No

1 (1 - 101865) 2 (2 - 101866) 3 (3 - 101867) 4 (4 - 101868)

should_flex_ext_paon {Horizontal Radiobutton}

Abduction of both arms

1 = symmetric
2 = deficit in affected
3 = deficit in unaffected

1 (1 - 101865) 2 (2 - 101866) 3 (3 - 101867)

should_abduc {Horizontal Radiobutton}

Extent of abduction deficit

1 = symmetric
2 = deficit in affected
3 = deficit in unaffected

1 (1 - 101865) 2 (2 - 101866) 3 (3 - 101867)

should_abduc_def {Horizontal Radiobutton}

Was there any pain while examining abduction of the arms

1 = In affected limb
2 = in other limb
3 = both
4 = No

1 (1 - 101865) 2 (2 - 101866) 3 (3 - 101867) 4 (4 - 101868)

should_abduc_pain {Horizontal Radiobutton}

External rotation of shoulder (90° flexion in elbow)

1 = symmetric
2 = deficit in affected
3 = deficit in unaffected

1 (1 - 101865) 2 (2 - 101866) 3 (3 - 101867)

should_ext_rot {Horizontal Radiobutton}

Extent of external rotation deficit (degrees)

°

should_ext_rot_def {Number 3,0}

Internal rotation (90° flexion in elbow, placement of hand along the vertebrae)

1 = symmetric
2 = deficit in affected
3 = deficit in unaffected

1 (1 - 101865) 2 (2 - 101866) 3 (3 - 101867)

should_int_rot {Horizontal Radiobutton}

Extent of internal rotation deficit (difference of thumb location in cm)

°

should_int_rot_def {Number 3,0}

Was there any pain while examining rotation of the arms

1 = In affected limb
2 = in other limb
3 = both
4 = No

1 (1 - 101865) 2 (2 - 101866) 3 (3 - 101867) 4 (4 - 101868)

should_rot_pain {Horizontal Radiobutton}

Notes shoulder examination

optional

notes_should {Textarea 10,100}

{ IASAlternateStandardGroup: .. Standard - vertical, alternative layout }

ELBOW

ELBOW

Query

Tested?

yes (1 - 101864) no (2 - 101747)

tested_elb {Horizontal Radiobutton}

Flexion
1 = symmetric
2 = deficit in affected
3 = deficit in unaffected

1 (1 - 101865) 2 (2 - 101866) 3 (3 - 101867)

elb_flex {Horizontal Radiobutton}

Extent of flexion deficit

°

elb_flex_def {Number 3,0}

Extension
1 = symmetric
2 = deficit in affected
3 = deficit in unaffected

1 (1 - 101865) 2 (2 - 101866) 3 (3 - 101867)

elb_ext {Horizontal Radiobutton}

Extent of extension deficit

°

elb_ext_def {Number 2,0}

Inspection: Flexion contracture
1 = No contracture
2 = flexion contracture in affected arm
3 = flexion contracture in unaffected arm

1 (1 - 101865) 2 (2 - 101866) 3 (3 - 101867)

elb_flex_contr {Horizontal Radiobutton}

Was there any pain while examining flexion and extension of the shoulders
1 = In affected limb
2 = in other limb
3 = both
4 = No

1 (1 - 101865) 2 (2 - 101866) 3 (3 - 101867) 4 (4 - 101868)

elb_flex_ext_pain {Horizontal Radiobutton}

Pronation in both arms
1 = symmetric
2 = deficit in affected
3 = deficit in unaffected

1 (1 - 101865) 2 (2 - 101866) 3 (3 - 101867)

elb_pron {Horizontal Radiobutton}

Extent of pronation deficit

°

elb_pron_def {Number 3,0}

Supination in both arms
1 = symmetric
2 = deficit in affected
3 = deficit in unaffected

1 (1 - 101865) 2 (2 - 101866) 3 (3 - 101867)

elb_sup {Horizontal Radiobutton}

Extent of supination deficit

°

elb_sup_def {Number 3,0}

Was there any pain while examining pro- and supination in elbows

1 = In affected limb
2 = in other limb
3 = both
4 = No

1 (1 - 101865) 2 (2 - 101866) 3 (3 - 101867) 4 (4 - 101868)

elb_pron_pain {Horizontal Radiobutton}

Notes elbow examination

optional

notes_elb {Textarea 10,100}

{ IASAlternateStandardGroup: .. Standard - vertical, alternative layout }

WRIST

WRIST

Query

Tested?

yes (1 - 101864) no (2 - 101747)

tested_wrist {Horizontal Radiobutton}

Palmar flexion in both wrists

1 = symmetric
2 = deficit in affected
3 = deficit in unaffected

1 (1 - 101865) 2 (2 - 101866) 3 (3 - 101867)

wrist_flex_palm {Horizontal Radiobutton}

Extent of palmar flexion deficit

°

wrist_flex_palm_def {Number 3,0}

Dorsal flexion in both wrists

1 = symmetric
2 = deficit in affected
3 = deficit in unaffected

1 (1 - 101865) 2 (2 - 101866) 3 (3 - 101867)

wrist_flex_dors {Horizontal Radiobutton}

Extent of dorsal extension deficit

°

wrist_flex_dors_def {Number 2,0}

Was there any pain while examining flexion and extension of the hands

1 = In affected limb
2 = in other limb
3 = both
4 = No

1 (1 - 101865) 2 (2 - 101866) 3 (3 - 101867) 4 (4 - 101868)

wrist_flex_ext_pain {Horizontal Radiobutton}

Ulnar abduction in both wrists

1 = symmetric
2 = deficit in affected
3 = deficit in unaffected

1 (1 - 101865) 2 (2 - 101866) 3 (3 - 101867)

wrist_uln_abd {Horizontal Radiobutton}

Extent of ulnar abduction deficit

°

wrist_uln_abd_def {Number 3,0}

Radial abduction in both wrists

1 = symmetric
2 = deficit in affected
3 = deficit in unaffected

1 (1 - 101865) 2 (2 - 101866) 3 (3 - 101867)

wrist_rad_abd {Horizontal Radiobutton}

Extent of radial abduction deficit

°

wrist_rad_abd_def {Number 3,0}

Was there any pain while examining the abduction of the hands

1 = In affected limb
2 = in other limb
3 = both
4 = No

1 (1 - 101865) 2 (2 - 101866) 3 (3 - 101867) 4 (4 - 101868)

wrist_abd_pain {Horizontal Radiobutton}

Notes wrist examination

optional

notes_wrist {Textarea 10,100}

{ IASHeadline: .... Headline }

Comments general

{ IASAlternateStandardGroup: .. Standard - vertical, alternative layout }

Comments

Query

optional

comments {Textarea 10,100} **"Comments general"**

---

leg_axis_stand_det

1. 1 (1 - 101865)
2. 2 (2 - 101866)
3. 3 (3 - 101867)
4. 4 (4 - 101868)
5. 5 (5 - 103699)
6. 6 (6 - 103700)
7. 7 (7 - 103701)
8. 8 (8 - 103702)
9. 9 (9 - 103703)
10. other (99 - 101752)

talocrural_det

1. 1 (1 - 101865)
2. 2 (2 - 101866)
3. 3 (3 - 101867)
4. 4 (4 - 101868)
5. 5 (5 - 103699)
10. other (99 - 101752)

pelvic_obl

1. 0 (0 - 103706)
2. 0-1 (1 - 103707)
3. 1-2 (2 - 103708)
4. 2-3 (3 - 103709)
5. 3-4 (4 - 103710)
6. 4-5 (5 - 103711)

leg_axis_walk_det

1. 1 (1 - 101865)
2. 2 (2 - 101866)
3. 3 (3 - 101867)
4. 4 (4 - 101868)
5. 5 (5 - 103699)
10. other (99 - 101752)

fem_tor_det

1. 1 (1 - 101865)
2. 2 (2 - 101866)
3. 3 (3 - 101867)
10. other (99 - 101752)

tib_tor_det

1. 1 (1 - 101865)
2. 2 (2 - 101866)
3. 3 (3 - 101867)
5. 4 (4 - 101868)
6. 5 (5 - 103699)
10. other (99 - 101752)

---

secuTrial® 5.5.0.12, 2019
