# Supplementary material for: Long-Term Outcomes of Acute Osteoarticular Infections in Children
Source: Front Pediatr. 2020 Nov 25;8:587740. doi: 10.3389/fped.2020.587740 (PMC7737431; doi:10.3389/fped.2020.587740)
Supplement: Supplementary file 5 [file Data_Sheet_5.PDF]

Supplementary Material 5. Disability scoring of upper and lower extremeties based on pain and physical activities

Modified after the PODCI (Pediatric Outcomes Data Collection Instrument)

Scoring parameter	Points	Total
**Lower Extremity**		
1) Current pain		0-5
Never	0	
less than once a month	1	
once a month	2	
once a week	3	
more than once a week	4	
Daily	5	
2) Short distance running		0-3
Yes, unrestricted	0	
Yes, slightly restricted	1	
Yes, severely restricted	2	
No	3	
3) Long distance running		0-3
Yes, easily	0	
Yes but a little hard	1	
Yes, but very hard	2	
No	3	
4) Walking > 100m		0-3
Yes, easily	0	
Yes but a little hard	1	
Yes, but very hard	2	
No	3	
5) Walking > 1000m		0-3
Yes, easily	0	
Yes but a little hard	1	
Yes, but very hard	2	
No	3	
6) Climbing stairs		0-3
Yes, easily	0	
Yes but a little hard	1	
Yes, but very hard	2	
No	3	
7) Walking uphill		0-3
Yes, easily	0	
Yes but a little hard	1	
Yes, but very hard	2	
No	3	

8) Walking downhill		0-3
Yes, easily	0	
Yes but a little hard	1	
Yes, but very hard	2	
No	3	
9) Ball games and sports		0-3
Yes, easily	0	
Yes but a little hard	1	
Yes, but very hard	2	
No	3	
10) Kneeling		0-3
Yes, easily	0	
Yes but a little hard	1	
Yes, but very hard	2	
No	3	
11) Sitting		0-3
Yes, easily	0	
Yes but a little hard	1	
Yes, but very hard	2	
No	3	
**Disability score lower extremity**	**Points**	
None	0	
Minor	1-11	
Moderate	12-23	
Severe	24-35	

Scoring parameter	Points	Total
**Upper extremity**		
12) Current pain		0-5
Never	0	
less than once a month	1	
once a month	2	
once a week	3	
more than once a week	4	
Daily	5	
13) Light work		0-3
Yes, easily	0	
Yes but a little hard	1	
Yes, but very hard	2	
No	3	
14) Heavy work		0-3
Yes, easily	0	

Yes but a little hard	1	
Yes, but very hard	2	
No	3	
15) Fine motor skills / working with tools		0-3
Yes, easily	0	
Yes but a little hard	1	
Yes, but very hard	2	
No	3	
16) Putting weight on the arms, e.g. plank position		0-3
Yes, easily	0	
Yes but a little hard	1	
Yes, but very hard	2	
No	3	
17) Overhead work		0-3
Yes, easily	0	
Yes but a little hard	1	
Yes, but very hard	2	
No	3	
**Disability score upper extremity**	**Points**	
No disability	0	
Minor disability	1-6	
Moderate disability	7-13	
Severe disability	14-20	

Questions 2-11 and 13-17: If questions was answered other than “Yes, easily”, points were not assigned if reason for limitation was associated with dislike of that activity.
